# Efficacy of psychosensory interventions for the management of pediatric pain, fear, and distress during emergency care: a systematic review and meta-analysis of randomized clinical trials

**DOI:** 10.3389/fped.2025.1654835

**Published:** 2026-01-07

**Authors:** Mariela Bustamante Fernández, Line Caes, José Iván Rossel, Genesis Díaz Díaz, Gabriela Ruiz Valenzuela, Scarlett Caroca Madariaga, Nicole Klein Vallecillo, Sofía Hidalgo Vilche, Valeska Tapia Espinoza, Mariana González Zamarin, Katherine Strasser

**Affiliations:** 1 Department of Pediatrics and Child Surgery, West Campus, Faculty of Medicine, Universidad de Chile, Santiago, Chile; 2 School of Psychology, Faculty of Social Sciences, Universidad de Chile, Santiago, Chile; 3 Division of Psychology, Faculty of Natural Sciences, University of Stirling, Stirling, United Kingdom; 4 School of Medicine, Universidad de Chile, Santiago, Chile; 5 School of Psychology, Pontificia Universidad Católica de Chile, Santiago, Chile

**Keywords:** pediatric emergency department, pediatric pain, fear, distress, psychosensory interventions, meta-analysis, RCT (randomised controlled trial)

## Abstract

The experience of invasive medical procedures (IMP) in pediatric emergency departments (PEDs) is often highly stressful for children and adolescents and potentially leads to short- and long-term biopsychosocial consequences. To address these challenges, psychological and sensory strategies have been developed to mitigate negative outcomes and enhance children's adaptive coping PEDs.

**Objective:**

To evaluate the efficacy of psychological and sensory interventions on pediatric pain, fear, and distress during PEDs.

**Methods:**

A systematic review and meta-analysis were conducted following the *PRISMA 2020 Statement* (PROSPERO registration: CRD42023403583). Searches were performed in PubMed, PsycInfo, CINAHL, SCOPUS, and Web of Science for studies published between January 2004 and September 2024. Randomized controlled trials involving children aged 2–18 years undergoing IMPs in PEDs. The qualitative synthesis and meta-analysis were performed using the Review Manager (RevMan) web versioN.Effect sizes were estimated using *Z*-scores (*Z*), *p*-values (*p*), and standardized mean differences (SMD).

**Results:**

A total of 1,796 records were retrieved (PubMed = 513, PsycInfo = 148, CINAHL = 516, SCOPUS = 183, Web of Science = 436); 45 were retained for the meta-analysis. Psychosensory interventions were grouped into seven subcategories: Somato-Sensory, Immersive Reality, Screen-Based, Toy Interaction, Social Interaction, Active Command, and Unisensory Distractions. The meta-analysis revealed significant reductions in both self-reported pain and fear (*Z* = 7.66/6.18, *p* < 0.01; SMD = −0.94/−1.30) and observed pain and fear (*Z* = 6.27/5.28, *p* < 0.01; SMD = −1.52/−1.77) across all intervention categories. The most effective interventions were pre-procedural informational videos (*Z* = 21.54/21.24, *p* < 0.01; SMD = −2.40/−2.18) and somato-sensory procedural distraction (*Z* = 5.37, *p* < 0.01; SMD = −1.08). Playful social interaction strategies (*Z* = 7.38, *p* < 0.01; SMD = −1.20), behavioral command (*Z* = 6.22/4.41, *p* < 0.01; SMD = −1.05/−0.87), and immersive reality (*Z* = 4.66, *p* < 0.01; SMD = −0.95) were also found to reduce observer-reported distress. However, a high degree of heterogeneity was observed in the results (*I*^2^ > 90%/98), which warrants a cautious interpretation.

**Discussion:**

Both pre-procedural preparation strategies and somatosensory procedural distractions are promising approaches for managing stressful experiences in PED settings. These approaches may address the sensory, cognitive, and emotional components of procedural pain, fear and distress. The evidence obtained could inform the development of clinical protocols aimed at optimizing children' experiences in PEDs and potentially minimizing long-term psychological and somatosensory consequences.

**Systematic Review Registration:**

https://www.crd.york.ac.uk/PROSPERO/display_record.php?ID=CRD42023403583, PROSPERO CRD42023403583.

## Introduction

Facing invasive medical procedures (IMPs) in a pediatric emergency department (PED) can be a stressful and potentially traumatic experience for children and adolescents (1–3), as it involves greater stressors compared to other healthcare contexts (4, 5). The structure and functioning of a PED are characterized by a crowded and fast-paced environment, unpredictability, and sensory overstimulation across admission, diagnosis, and treatment (6–11). This process is also frequently compounded by stressors related to the acute illness that prompted the visit, caregiver stress levels, and the procedural pain experience itself (12–18). Furthermore, IMPs in PEDs often involve high levels of anticipatory fear and procedural pain (19), which may increase behavioural distress, and in effect, child verbal and physical resistance, leading to higher stress for caregivers and healthcare personnel (19–21). High behavioral distress may also imply prolonged procedures and lower efficiency required in PEDs (22, 23).

Pain experiences, fear, and behavioral distress in PEDs not only impact the immediate experience of the pediatric patient but can also have long-term psychological and somatosensory consequences. Psychologically, there is a significant link between negative pre-hospital and hospital experiences and the development of negative pain memories, anxiety, depression, avoidance behaviors, unnecessary visits, and poor adherence to health advice (24–27). From a somatosensory perspective, neuroscience studies suggest that children may be at higher risk of developing central sensitization (28–30), leading to increased pain sensitivity, lowered pain thresholds, chronic pain, and lasting changes in pain pathways (28, 31–35). Therefore, it is essential to identify interventions that are both effective and efficient in PEDs to improve patient experiences and reduce immediate and long-term complications (36).

Some psychological and sensory interventions have proven effective in reducing pain, fear, and behavioral distress in outpatient IMPs (37). Sensory interventions involve the use of specific sensory stimuli (tactile, auditory, visual, proprioceptive, vestibular) to enhance sensory regulation and adaptive response to painful stimuli (38, 39). Psychological interventions integrate cognitive, emotional, behavioral, and social elements in the modulation of pain (40–42). Psychosensory interventions, in this context, refer to strategies that integrate both sensory stimulation and psychological regulation processes to influence pain perception and emotional responses. The theoretical and empirical foundations of how psychosensory approaches support a biopsychosocial modulation of pain are promising for advancing understanding of the mechanisms underlying their effectiveness (43, 44). Despite the growing use and evaluation of psychosensory approaches in PEDs, a comprehensive overview of their comparative effectiveness remains limited, which is crucial for appropriate evidence-based implementations within this complex setting. Hence, this systematic review and meta-analysis aims to: (1) analyze and summarize the current state-of-the-art evidence (acquired through randomized controlled trials [RCTs]) on the effectiveness of psychological and sensory interventions in managing pain, fear, and distress in children undergoing IMPs in PEDs; and (2) analyze the methodological rigor and quality of the RCTs included in the review.

## Materials and methods

### Procedure

The study was conducted following the Preferred Reporting Items for Systematic Reviews and Meta-Analyses (PRISMA, 2020) Statement to ensure transparency and thoroughness at all stages of the process (45). Searches were conducted over the last two decades (January 2004–September 2024), ensuring methodological and contextual relevance to current PEDs.

### Literature search

A systematic review of RCTs evaluating the effectiveness of psychological and/or sensory strategies to manage pediatric pain, fear, and behavioral distress was conducted, according to the pre-registered protocol on PROSPERO (CRD42023403583). The search terms focused on these primary outcomes, using combinations of descriptors such as “Children OR Adolescent,” “Psychological OR Sensory Intervention,” “Pain OR Fear OR Distress,” and “Emergency Department,” connected with the Boolean operator AND (see Supplementary Material 1). Boolean phrases were adapted to the thesauri of PubMed, CINAHL, PsycInfo, Scopus, and Web of Science databases. Gray literature was not included. However, backward and forward snowballing were performed on the reference lists and citations of the included studies to identify any eligible RCTs not captured in the primary database search.

These outcomes were prioritized because they represent the core emotional and behavioral manifestations of procedural stress in children, which are directly observable or self-reported and clinically relevant for evaluating the effectiveness of psychosensory interventions. Physiological or biological indicators (e.g., heart rate, respiratory rate, blood pressure, oxygen saturation, cortisol) were retained as complementary secondary outcomes when reported alongside emotional or behavioral measures, as they provide additional information about autonomic activation associated with procedural stress. However, these indicators were not included as specific search terms, as they were outside the scope of the main research question.

### Inclusion and exclusion criteria

Inclusion criteria were: (1) Studies published from 2004 onwards; (2) studies published in English or Spanish; (3) RCTs; (4) studies with at least one control group with usual care; (5) children aged 2–18 years; (6) studies evaluating a psychological, sensory, or mixed psychosensory intervention implemented within PED care; (7) PED as the setting intervention; (8) sample undergoing at least one IMPs for diagnosis or treatment purposes (e.g., intravenous line, blood sample, or injections); (9) studies evaluating pain, fear, or behavioral distress as outcomes assessed through self-report or observer-report.

Studies were excluded if: (1) children had a severe chronic diagnosis that could interfere with outcomes (e.g., cancer, cystic fibrosis); (2) studies were caregiver-focused only; (3) hospitalization or outpatient settings; (4) interventions applied only on the waiting room or post-discharge; (5) PED care without IMPs; (6) sample size below 30 children, and (7) studies focusing exclusively on physiological or biochemical parameters.

### Selection process

**Figure 1:**
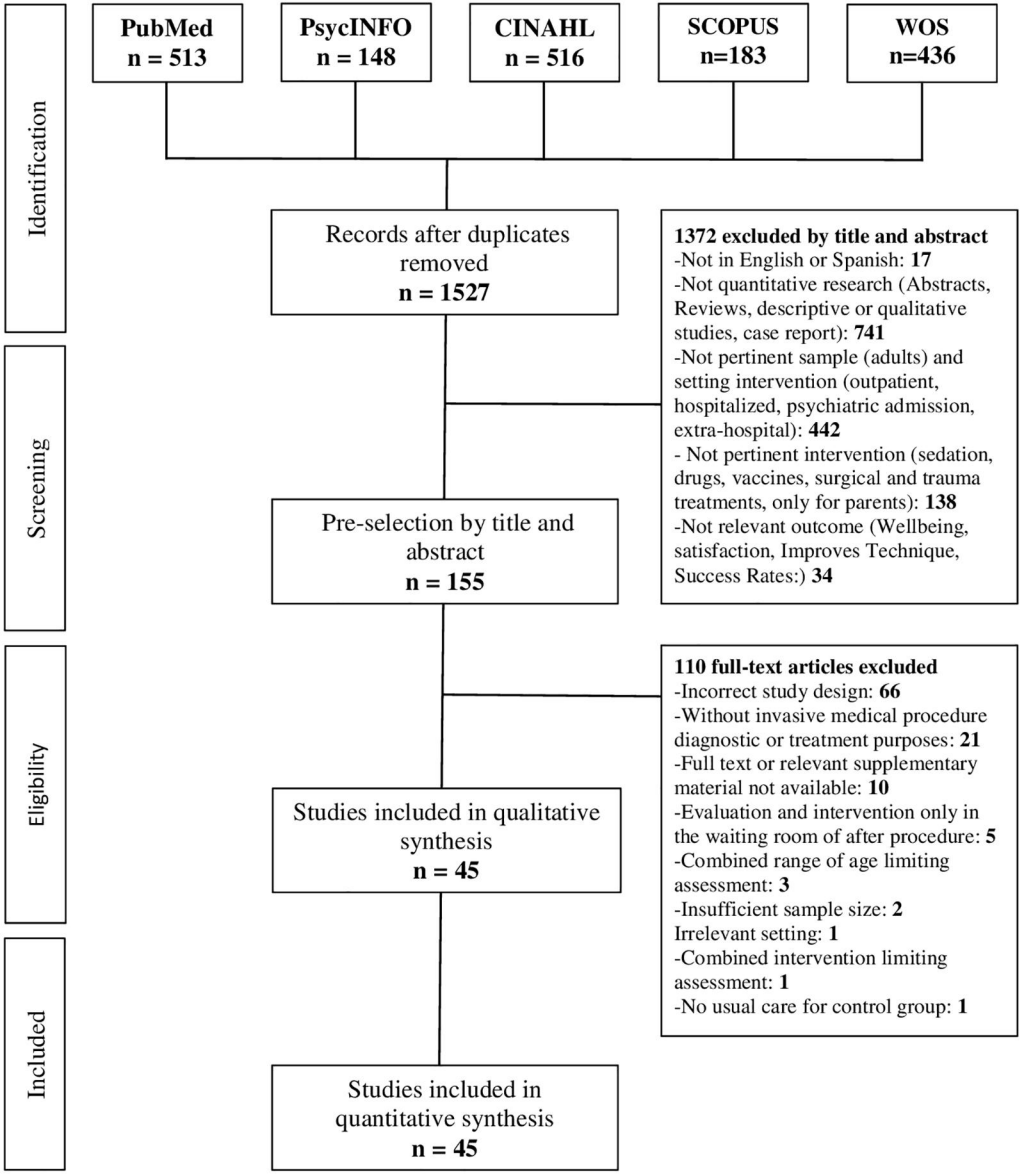
PRISMA flow diagram.

The article selection process is presented in the PRISMA flowchart (see Figure 1). Two reviewers (MB and VT) independently selected the articles meeting the eligibility criteria by first reviewing all identified titles and abstracts, and subsequently all full texts selected after the abstract selection. Discrepancies between reviewers were resolved by consensus.

### Data extraction and synthesis

Data extraction was performed independently by two reviewers (MB and GD) using a predefined extraction form, which included: study design, sample size, type of IMPs, participant demographic information (age, gender), details of interventions (type, duration, frequency, and timing), outcomes measured (self-reported, observed pain, fear, behavioral distress as well as biological markers), and statistical results (mean differences, standard deviations [SD], *p*-values, and confidence intervals [CI]) (see Table 1).

**Table 1:** Characteristics of included studies.

Author	Sample & Country	Objective	Main Intervention	Specific intervention	Timing	Control	Outcomes and Measured	Results
Akarsu, Semerci, and Kılınç, 2023 (65)	159 (5 to 12 years) M = 8.39/SD = 2.25 Boys: 49.7% Girls: 50.3%. Turkey	Compare the effects of watching cartoons with VR and tablet on pain, fear, and anxiety during venipuncture	Screen-Based and Immersive Reality	Video with VR Glasses (Group 1). Cartoon on Portable Device (Group 2)	Before and During	Usual procedure. Standard parental support.	Pain (WB-FACES) and Fear (CFS). Self-Reported and Observer (Caregiver and Nurse).	VR and Cartoon were effective in reducing self-reported pain (*M* = 0.79/2.03, *p* < 0.01) and observed pain (*M* = 0.87/2.07, *p* < 0.01), as well as self-reported fear (*M* = 0.47/0.94, *p* < 0.01) and observed fear (*M* = 0.44/0.94, *p* < 0.01), compared control group (*M* = 4.45–4.54).
Ali et al., 2021a (81)	86 (6 to 11 years) M = 9/SD = 2.22 Boys: 60.5% Girls: 48.8%. Canada	Evaluate the effectiveness of a humanoid robot to reduce stress and pain during intravenous cannulation	Social Interaction	Robot Coach (Group 1).	Before, During, and After	Usual procedure. Standard parental support.	Self-Reported Pain (FPS-R), Behavioral Distress (OSBD-R) Biological Marker: HR.	Robot intervention showed a significant difference compared control in distress (*M* = 0.37/0.74, *p* < 0.03), but not for pain (*M* = 2/4, *p* = 0.10), or heart rate (*M* = 120/119, *p* = 0.86).
Ali et al., 2021b (66)	86 (6 to 11 years) M = 7/SD = 2.96 Boys: 52.9% Girls: 47.1%. Canada	Compare the effectiveness of using an iPad vs. standard care on pain and distress during intravenous cannulation	Screen-Based	Cartoon on Portable Device (Group 1).	Before and during	Usual procedure. Standard parental support. The nurse used usual calming verbalization	Self-Reported Pain (FPS-R), Behavioral Distress (OSBD-R), Biological Marker: HR.	No significant differences were found in pain (Control/iPad: *M* = 3.0/3.0, *p* = 0.35), distress (Control/iPad: *M* = 2.09/1.34, *p* = 0.09), or heart rate (iPad/Control: *M* = 119/116, *p* = 0.44).
Arikan and Esenay, 2020 (71)	216 (6 to 12 years) M = 9.2/SD = 1.58 Boys: 50% Girls: 50%. Turkey	Examine the effects of active and passive distraction on pain, fear, and anxiety during blood sampling	Toy-Based Distractions	Interaction with Rotating Toy (Group 1) and Musical Bracelet (Group 2)	During	Usual procedure. Standard parental accompaniment	Self-Reported Pain (WB-FACES/VAS), and Fear (CFS).	Both the active and passive distraction groups significantly reduced pain for VAS (Active/Passive/Control: *M* = 1.50/1.97/3.79, *p* < 0.01), WB-FACES (Active/Passive/Control: *M* = 2.60/3.30/7.33, *p* < 0.01), and fear (Active/Passive/Control: *M* = 1.63/2.09/3.9, *p* < 0.01).
Baxter et al., 2011 (47)	81 (4 to 18 years) M = 10.01/SD = 0.95 Boys: 51.9% Girls: 48.1%. USA	Compare a reusable cold and vibration device with standard care to alleviate pain from pediatric venous access	Somato-Sensory	Buzzy or Vibration (Group 1).	During	Usual procedure. Standard parental accompaniment	Pain self-reported and observed (FPS-R), Behavioral Distress (OSBD-R).	Significant differences between Buzzy and control for self-reported pain (*M* = 1/2, *p* *=* 0.029), observed pain (*M* = 2/4, *p* < 0.01), and behavioral distress (*M* = 2/4, *p* = 0.036).
Bourdier et al., 2021 (91)	607 (1,5 to 6 years) M = 3.7/SD = 1.12 Boys: 52% Girls: 48%. France	Evaluate differences in pain-related behavior during cannulation between the buzzy device and standard care	Somato-Sensory	Buzzy or Vibration (Group 1).	During	Usual procedure. Standard parental accompaniment. EMLA patch	Behavioral Distress (CHEOPS).	Control group had lower distress scores than those in the Buzzy group (Control/Buzzy: *M* = 7.2/8.5, *p* < 0.01).
Can et al., 2024 (74)	160 (4 to 10 years) M = 6.85/SD = 2.01 Boys:47.5% Girls: 52.5%. Turkey	Evaluate the effect of Veinlite PEDI2 (VID) and VR on emotional behavior, pain, fear, and anxiety	Unisensory Distractions Immersive Reality	VID (Group 1), Video with VR Glasses (Group 2), and Video VR Glasses + VID (Group 3)	Before, during, and after	Usual procedure. Standard parental accompaniment	Self-reported Pain (WB-FACES/CAS) and Fear (CFS).	Significant differences were found for WB-FACES pain (Control/VID/VR/VID + VR: *M* = 6.0/6.0/4.4/4.6, *p* = 0.004) and CAS pain (Control/VID/VR/VID + VR: *M* = 6.8/6.8/5.7/4.7, *p* < 0.001). No significant differences were found for fear (Control/VID/VR/VID + VR: *M* = 2.4/2.2/1.8/1.8, *p* = 0.09).
Cavender et al., 2004 (82)	43 (4 to 11 years) M = 7.89/SD = 1.74 Boys:44% Girls: 56%. USA	Determine the effectiveness of parental involvement using positioning and distraction to reduce pain, fear, and distress during venipuncture	Social Interaction	Parental Positioning and Distraction (Group 1).	Before, during, and after	Usual procedure. Standard parental accompaniment.	Pain (WB-FACES) and Fear (GFS) self-reported Behavioral Distress (PBCL).	The intervention group did not show significant differences compared the control in pain (*M* = 2.74/2.3, *p* = 0.68), fear (*M* = 2.74/2.15, *p* = 0.058), or distress (*M* = 12/16, *p* = 0.13).
Celik et al., 2023 (48)	96 (7 to 15 years) M = 10.67/SD = 2.86 Boys: 53.1% Girls: 46.9%. Turkey	Determine the effect of cold spray and ice applied during venipuncture on the level of fear and pain in children	Somato-Sensory	Cold Spray (Group 1) and Ice Gel (Group 2)	Before and during	Usual procedure. Standard parental accompaniment	Pain (VAS) and Fear (CFS) Self-reported and observed.	Cold Spray significantly reduced both pain and fear, both self-reported and observed, compared to Cuidado Usual (Pain: *M* = 2.63/7.49, *p* < 0.0001; Fear: *M* = 1.78/3.81, *p* < 0.01). Ice Gel also reduced pain and fear, but only self-reported fear reached statistical significance (Pain: *M* = 6.75/7.49, *p* = 0.10; Fear: *M* = 3.31/3.81, *p* = 0.045)
Ceylan and Erkut, 2023 (89)	80 (3 to 6 years) M = 4.27/SD = 1.11 Boys: 52.5% Girls: 47.5%. Turkey	Determine the effect of distraction with a finger puppet during venous blood sampling on children’s pain and emotional manifestation	Social Interaction	Finger Puppet (Group 1).	Before and during	Usual procedure. Standard parental accompaniment	Behavioral Distress (CEMS)	Distress measures were statistically lower in the experimental group (Puppet/Control: *M* = 9.12/15.62, *p* < 0.001).
Chan et al., 2019 (75)	252 (4 to 11 years) M = 7.91/SD = 1.03 Boys: 56% Girls: 44%. Australia	Evaluate the efficacy and safety of VR distraction for needle pain in the emergency department and outpatient settings	Immersive Reality	Video with VR Glasses (Group 1).	Before and during	Usual procedure. Standard parental accompaniment. age-appropriate distractions	Self-reported Pain (FPS-R) and Fear (VAT).	VR significantly reduced both pain (VR/Control: *M* = 2/4.39, *p* < 0.01) and distress (VR/Control: *M* = 2/4.33, *p* < 0.01) compared to control.
Chen et al., 2020 (76)	136 (7 to 12 years) M = 9.15/SD = 1.70 Boys: 56.6% Girls: 43.4%. Taiwan	Examine the effects of VR on pain and fear in school-aged children during intravenous injection	Immersive Reality	Video with VR Glasses (Group 1).	During	Usual procedure. Standard parental accompaniment	Pain (WB-FACES), Fear (CFS), both self-reported and observed.	VR intervention significantly reduced for fear observed VR/Control: *M* = 1.46/2.09, *p* = 0.043) and self-reported (VR/Control: *M* = 1.32/1.78, *p* = 0.043), and for pain observed (VR/Control: *M* = 3.28/4.29, *p* = 0.031) and self-reported (VR/Control: *M* = 3.35/4.35, *p* = 0.031)
Daihimfar et al., 2024 (61)	180 (3 to 6 years) M = 4.36/SD = 0.66 Boys: 48.9% Girls: 51.1%. Iran	Compare the effects of acupressure and music therapy on venipuncture pain intensity in children	Unisensory Distractions Somato-Sensory	Music Therapy (Group 1) and Acupressure (Group 2).	Before and during	Usual procedure. Standard parental accompaniment	Self-reported Pain (Oucher Scale).	The lowest mean pain score was in the music therapy group, and the highest in the control group (Music/Acupressure/Control: *M* = 3.32/4.82/8.32, *p* < 0.001).
Downey et al., 2012 (67)	100 (3 to 18 years) M = 8.57/SD = 4.42 Boys: 38.4% Girls: 61.6%. USA	Determine if watching cartoons in an acute care setting reduces pain perception in children	Screen-Based	Cartoons on Tablet or Portable DVD Player (Group 1).	During	Usual procedure. Standard parental accompaniment	Self-reported Pain (FPS-R).	Pain score differences during the procedure were minimal (Cartoons: *M* = 6.42 vs. Control: *M* = 6.47, *p* = 0.93).
Dumoulin et al., 2019 (68)	177 (8 to 17 years) M = 13.35/SD = 2.96 Boys: 64.4% Girls: 35.6%. Canada	Compare the efficacy of VR during a medical procedure with two other conditions: watching television and CLS	Immersive Reality Screen-Based	Video Game with VR (Group 1) and Cartoons on Tablet or Portable DVD Player (Group 2)	During	Usual procedure. Standard parental accompaniment. The CLS program	Self-reported Pain and Fear (VAS).	VR group was effective in reducing pain intensity (Cartoon: *M* = 35.43, Control: *M* = 25.33, VR: *M* = 21.75) and fear of pain (Cartoon: *M* = 35.42, Control: *M* = 29.33, VR: *M* = 19.75), with statistically significant results (*p* < 0.05).
Düzkaya et al., 2020 (62)	477 (6 to 12 years) M = 8.8/SD = 2.05 Boys: 51.6% Girls: 48.4%. Turkey	Compare the effects of watching a cartoon and an informational video on children’s pain and fear levels	Screen-Based	Cartoons on Tablet or Portable DVD Player (Group 1) and Informational Video (Group 2)	Before and during	Usual procedure. Standard parental accompaniment	Pain (WB-FACES), Fear (CFS), both self-reported and observed, Biological Markers: SpO2, HR, BP.	Informational video and cartoon groups had lower pain (*M* = 0.09/0.30 vs. 4.14, *p* = 0.01), fear (*M* = 0.05/0.32 vs. 3.41, *p* = 0.01), HR (*M* = 110.90/109.79 vs. 132.54, *p* = 0.01), and BP (*M* = 62.71/62.14 vs. 70.34, *p* = 0.01). No significant differences in SpO2 (*M* = 99.01/98.98 vs. 98.92, *p* = 0.168).
Düzkaya et al., 2024 (49)	177 (6 to 12 years) M = 8.73/SD = 2.02 Boys: 48% Girls: 52%. Turkey	Determine the effects of ShotBlocker and the Helfer Skin Puncture technique on children’s pain and fear during intramuscular injection	Somato-Sensory	ShotBlocker (Group 1) and Helfer Skin Puncture (Group 2)	During	Usual procedure. Standard parental accompaniment	Pain (WB-FACES) Fear (CFS), both self-reported and observed.	Shockblocker had lowest scores for observed fear and pain (*M* = 2.22/2.21), followed by Acupressure (*M* = 2.63/ 2.62), and control (*M* = 2.92/ 2.91). Same patron for self-reported outcomes (*M* = 2.56/ 2.68 vs. 2.97/3.08 vs. 3.34/3.46) with significant differences (*p* < 0.05).
Farion et al., 2008 (50)	80 (6 to 12 years) M = 9.4/SD = 2.04 Boys: 52.5% Girls: 47.5%. Canada	Determine whether a new product, Pain Ease, would reduce pain during intravenous cannulation in children	Somato-Sensory	Cold Spray (Group 1).	Before	Standard parental accompaniment. Placebo aerosol. CLS distraction	Self-reported and Observed Pain (VAS). Caregivers, Nurses, and CLS.	Cold Spray showed lower pain compared to usual care, both in observed pain (*M* = 0.96 vs. 1.38, *p* < 0.01) and self-reported pain (*M* = 36.9 vs. 56.1, *p* < 0.01).
Felluga et al., 2016 (83)	40 (4 to 11 years) M = 9.0/SD = 2.56 Boys: 67.5% Girls: 32.5%. Italy	Evaluate if the presence of medical clowns can reduce children’s anxiety and pain during painful procedures	Social Interaction	Medical Clowns (Group 1).	Before and during	Usual procedure. Standard parental accompaniment. Nurse distraction	Self-reported Pain (NRS/WB-FACES), Fear (CAPS).	No significant differences were found in pain between the control group (*M* = 5 vs. 5.5, *p* = 0.183). However, the observed fear was significantly lower in the Clown group (*M* = 1 vs. 2.22, *p* = 0.0041).
Girgin et al., 2020 (51)	90 (6 to 12 years) M = 9.3/SD = 1.5 Boys: 50% Girls: 50%. Turkey	Compare the effects of ShotBlocker and Buzzy methods on pain, fear, and parental satisfaction during intramuscular injection	Somato-Sensory	Buzzy or Vibration (Group 1) and ShotBlocker (Group 2)	Before and during (Group 1), During (Group 2)	Usual procedure. Standard parental accompaniment	Pain (WB-FACES), Fear (CFS), both self-reported and observed.	Buzzy and ShotBlocker significantly reduced pain and fear. For observed and self-reported pain (Buzzy/ShotBlocker/Control: *M* = 0.2/1.23/2.99; *M* = 0.23/1.23/3.0, *p* *<* 0.01). For observed and self-reported fear (Buzzy/ShotBlocker/Control: *M* = 0.25/1.22/3.03; *M* = 2.40/2.47/2.33, *p* *<* 0.01)
Goktas and Avci, 2023 (72)	144 (7 to 12 years) M = 9.5/SD = 1.51 Boys: 50% Girls: 50%. Turkey	Determine the effects of visual and/or auditory distraction techniques during invasive procedures on pain, anxiety, and medical fear	Toy-Based Distractions Unisensory Distractions Immersive Reality	Kaleidoscope (Group 1), Listening to Music (Group 2), and Video with VR Glasses (Group 3)	Before, during, and after	Usual procedure. Standard parental accompaniment	Pain (WB-FACES), Fear (CFS), self-reported.	Pain levels were significantly lower in the intervention groups (Kaleidoscope/Music/VR/Control: *M* = 0.67/0.64/0.42/2.72, *p* < 0.001), as well as in fear scores (Kaleidoscope/Music/VR/Control: *M* = 41.86/40.97/42.08/49.47, *p* < 0.01).
Goldman and Behboudi, 2020 (77)	70 (6 to 16 years) M = 9.23/SD = 4.26 Boys: 54.5% Girls: 45.5%. Canada	Explore the role of VR in reducing pain and anxiety during intravenous catheterization, compared to standard treatment	Immersive Reality	Video with VR Glasses (Group 1)	Before, during, and after	Usual procedure. Standard parental accompaniment	Self-reported Pain (FPS-R) and Fear (VSAS)	VR group reported statistically significant lower pain scores compared to the control group (*M* = 2/4, *p* < 0.01). As for distress, both groups reported similar levels of distress (*M* = 2.4/2.4, *p* *=* 0.51).
Haidar et al., 2024 (52)	300 (2 to 14 years) M = 6.83/SD = 3.01 Boys: 49.0% Girls: 51.0%. Qatar	Evaluate the effect of the Buzzy device on pain and anxiety compared to EMLA cream during intravenous cannulation or venipuncture	Somato-Sensory	Buzzy or Vibration (Group 1)	Before and during	Usual procedure. Standard parental accompaniment. Topical EMLA	Self-reported Pain (FPS-R), Behavioral Distress (FLACC).	Mean self-reported pain scores were lower in the EMLA control group than Buzzy (*M* *=* 2.58/1.67; *p* *=* *0.062*), but it was not statistically significant. In contrast, children in the Buzzy group exhibited significantly higher distress than controls (*M* *=* 1.99 vs. 0.88; *p* *=* *< 0.01*).
Halal et al., 2022 (53)	120 (3 to 6 years) M = 4.39/SD = 0.97 Boys: 55.0% Girls: 45.0%. Iraq	Study the effect of the Buzzy device and whistling during blood sample collection on children’s pain and fear	Somato-Sensory Active Command	Buzzy or Vibration (Group 1) and Whistling (Group 2)	Before and during	Usual procedure. Standard parental accompaniment	Pain (WB-Faces), Fear (CMFS), both self-reported.	Pain showed statistically significant differences (Buzzy/Whistling/Control: *M* = 1.85/2.85/3.77, *p* < 0.01). as well as fear scores (Buzzy/Whistling/Control: *M* = 4.40/5.45/19.77, *p* < 0.01).
Hartling et al., 2013 (73)	42 (3 to 11 years) M = 5.92/SD = 3.29 Boys: 66.7% Girls: 33.3%. Canada	Compare music with standard care for managing pain and stress	Unisensory Distractions	Listening to Music (Group 1)	Before, during, and after	Usual procedure. Standard parental accompaniment	Self-reported Pain (FPS-R), Behavioral Distress (OSBD-R). Biological Marker: HR.	No significant difference was observed for pain (Control/Music: *M* = 4/2, *p* = 0.22), distress (*M* = 3.35/1.74, *p* = 0.27), and HR (*M* = 128.7/126.3, *p* = 0.72)
Karaca and Guner, 2022 (86)	60 (4 to 6 years) M = 4.93/SD = 0.88 Boys: 48.3% Girls: 51.7%. Turkey	Analyze the effect of a musical and moving toy distraction method on fear and anxiety	Toy-Based Distractions	Musical and Dancing Toy (Group 1)	Before and during	Usual procedure. Standard parental accompaniment	Self-reported and Observed Fear (CFS). Biological Marker: SpO2, HR.	There were no statistically significant differences in self-reported (Musical toy/Control: *M* = 2.97/2.87, *p* = 0.58) and observer fear (*M* = 2.82/2.69, *p* = 0.64). Neither in SpO2 (*M* = 96.86/97.30, *p* = 0.43, or HR (*M* = 125.79/133.06, *p* = 0.12). However, HR was significantly increased post-procedure (*M* = 126.77/111.67, *p* < 0.01).
Lee et al., 2022 (63)	88 (6 months to 4 years) M = 1.92/SD = 1.70. Boys: 60.2% Girls: 39.8%. Canada	Determine whether a VR environment reduces distress among children during IV catheter placement compared to standard care	Screen-Based	Ceiling Virtual Reality Display (Group 1)	Before and during	Usual procedure. Standard parental accompaniment	Behavioral Distress (FLACC), Observed Pain (VAS) and Fear (VAS).	There were no statistically significant differences for distress levels (Control/VR screen: *M* = 5.8/5.1, *p* = 0.28). In contrast, observed fear (Control/VR screen: *M* = 7.0/5.0, *p* < 0.01), and pain (Control/VR screen: *M* = 8.0/5.0, *p* < 0.01), showed significant differences.
Lilik et al., 2017 (87)	57 (3 to 6 years) M = 4.33/SD = 1.12 Boys: 57.9% Girls: 42.1%. Indonesia	Evaluate the effectiveness of cartoon-patterned clothing and bubbles for alleviating pain and anxiety during venipuncture	Unisensory Distractions Active Command	Cartoon-patterned Clothes (Group 1) and Blowing Bubbles (Group 2)	During	Usual procedure. Standard parental accompaniment	Behavioral Distress (FLACC), Fear Self-Reported (CFS).	Significant differences were observed in self-reported fear and an approach to significance for distress levels. Mean scores for fear were Control/Blowing Bubbles/Simple Visual: *M* = 3/1/2, *p* < 0.01/0.03). For distress, the scores were *M* = 5.79/4.11/4.32 (*p* = 0.02/0.06).
Miguez-Navarro, 2016 (69)	140 (3 to 11 years) M = 6.87/SD = 2.54 Boys: 57.9% Girls: 42.1%. Spain	Investigate the efficacy of video distraction to reduce anxiety and pain in children undergoing venipuncture	Screen-Based	Cartoons on Tablet or Portable DVD (Group 1)	Before and during	Usual procedure. Parental presence was not allowed in the study group	Self-reported Pain (WB-FACES/NRS), Behavioral Distress (GDS) Biological Marker: HR.	Significantly lower levels of distress in the video distraction group (Video/Control: *M* = 1.76/2.80, *p* < 0.001), as well as in pain (*M* = 3.18/5.74, *p* < 0.001). Additionally, HR was significantly higher in the control group (*p* = 0.01).
Miller et al., 2016 (64)	98 (3 to 12 years) M = 6.73/SD = 2.71 Boys: 49.0% Girls: 51.0%. Australia	Examine which of the interventions most effectively reduces pain and distress in children during IV cannulation	Screen-Based	PSP-VG (Group 1), Ditto-D (Group 2), Ditto-P (Group 3), Ditto-C (Group 4)	Before, during, and after	Usual procedure. Standard parental accompaniment	Self-reported Pain (WB-FACES) and Observer-reported Pain (VAS), Behavioral Distress (FLACC).	Significant reductions in pain and distress only for observed measures. Self-reported pain were: Control/PSP/Ditto-D/Ditto-PP/Ditto-C: *M* = 3.15/2.15/2.30/3.11/2.53 (*p* = 0.29). Observer-rated pain (*M* = 6.51/3.79/4.28/4.84/3.51 (*p* = 0.03). Distress: *M* = 4.90/1.95/2.60/3.21/3.32 (*p* = 0.09).
Oluc et al., 2024 (80)	111 (6 to 12 years) M = 6.73/SD = 2.71 Boys: 49.0% Girls: 51.0%. Turkey	Evaluate the effects of two distraction methods on reducing pain and fear during phlebotomy	Active Command	Blowing Bubbles (Group 1) and Stress Balls (Group 2)	Before and during	Usual procedure. Standard parental accompaniment	Fear (CFS), Pain (WB-FACES), both self-reported and observed.	Significant differences were found in self-reported fear (Bubbles/Stress Balls/Control: *M* = 0.89/1.4/2.49, *p* < 0.001) and pain levels (*M* = 2.59/2.97/5.84, *p* < 0.001).
Osmanlliu et al., 2019 (78)	63 (7 to 17 years) M = 11.7/SD = 2.95 Boys: 38.7% Girls: 61.3%. Canada	Study the feasibility and acceptability of VR distraction for patient comfort during intravenous procedures	Immersive Reality	VR Video Game (Group 1)	Before and during	Usual procedure. Standard parental accompaniment	Self-reported Pain (NRS), and Fear (CFS), Behavioral Distress (PBCL).	No significant differences were found in pain scores (VR/Control: *M* = 3.5/3.25, *p* = 0.75) or fear (1/1.5, *p* = 0.30). However, the VR group showed significantly less distress compared to control (*M* = 8.5/11.5, *p* = 0.002).
Rimon et al., 2016 (84)	53 (2 to 15 years) M = 6.19/SD = 3.09 Boys: 58.5% Girls: 41.5%. Israel	Investigate whether clown medical interventions reduce distress and have an effect on cortisol during venipuncture	Social Interaction	Medical Clowns (Group 1)	Before, during, and after	Usual procedure. Standard parental accompaniment	Self-reported Pain (VAS). Biological Markers: Plasma Cortisol	The intervention with clowns significantly reduced pain scores (Clowns: *M* = 2.2; Control: *M* = 7.5, *p* = 0.001). No significant differences were observed in plasma cortisol levels (Clowns: *M* = 16.4; Control: *M* = 18.3, *p* = 0.65).
Schlechter et al., 2021 (79)	115 (4 to 17 years) M = 11/SD = 4.2 Boys: 47.8% Girls: 52.2%. USA	Evaluate the efficacy of VR distraction to increase the success of the first intravenous attempt	Immersive Reality	VR Video Game (Group 1)	Before and during	Usual procedure. Standard parental accompaniment. and distraction	Pain (FPS-R), Fear (LTAS) both self-reported and observed (Caregivers)	There were no significant differences for self-reported/observer pain (VR: *M* = 4/4; Control: *M* = 4/4, *p* = 0.37/0.45). For self-reported/observer fear (VR: *M* = 1/1; Control: *M* = 1/2, *p* = 0.79/0.63).
Semerci et al., 2023 (54)	161 (5 to 12 years) M = 8.34/SD = 2.16 Boys: 51.6% Girls: 48.4%. Turkey	Evaluate the efficacy of Buzzy and Cold Spray in reducing pain, anxiety, and fear during venipuncture	Somato-Sensory	Buzzy (Group 1) and Cold Spray (Group 2)	Before and during	Usual procedure. Standard parental accompaniment	Pain (WB-FACES), Fear (CFS), both self-reported and observed (Caregivers and health workers)	Cold Spray and Buzzy significantly reduced both observed and self-reported pain and fear (*p* < 0.001). For fear, control (*M* = 3.5/3.42), Cold Spray (*M* = 0.95/0.89), and Buzzy (*M* = 1.26/1.06). For pain, control (*M* = 5.91/6.31), Cold Spray (*M* = 1.09/1.10), and Buzzy (*M* = 1.46/1.48).
Şen et al., 2024 (88)	102 (7 to 12 years) M = 9.11/SD = 1.67 Boys: 62.7% Girls: 37.3%. Turkey	Evaluate the effect of VR glasses during IV catheter insertion on children’s emotional responses	Immersive Reality	Video with VR Glasses (Group 1)	During	Usual procedure. Standard parental accompaniment	Behavioral Distress (CEMS).	The VR group scored significantly lower than the control group (VR: *M* = 6.21; Control: *M* = 10.54, *p* < 0.01).
Sivri et al., 2019 (55)	150 (7 to 12 years) M = 8.92/SD = 1.88 Boys: 54% Girls: 46%. Turkey	Investigate the effect of Buzzy and ShotBlocker on reducing pain from intramuscular penicillin injections in children	Somato-Sensory	Buzzy or Vibration (Group 1) and ShotBlocker (Group 2)	Before and during	Usual procedure. Standard parental accompaniment	Pain self-reported (VAS/FPS-R).	Pain scores were significantly lower in Buzzy and ShotBlocker for both VAS/FPS-R (Control: *M* = 7.34/7.36; ShotBlocker: *M* = 6.36/6.24; Buzzy: *M* = 3.68/3.64, *p* < 0.01).
Sivri et al., 2023 (56)	242 (9 to 12 years) M = 10.40/SD = 1.40 Boys: 45.9% Girls: 54.9%. Turkey	Compare the effectiveness of Buzzy, ShotBlocker, and Distraction Cards in reducing pain and anxiety during venous blood sampling in children	Somato-Sensory	Buzzy or Vibration (Group 1), ShotBlocker (Group 2), and Distraction Cards (Group 3)	Before and during	Usual procedure. Standard parental accompaniment	Pain self-reported (VAS/FPS-R).	Pain was significantly higher in the control group, for VAS and FPS-R (Control: *M* = 2.98/2.98; ShotBlocker: *M* = 1.77/1.77; Buzzy: *M* = 1.07/1.07; Cards: *M* = 1.12/1.13, *p* = *< 0.01*).
Stevenson et al., 2005 (90)	149 (2 to 16 years) M = 8.44/SD = 4.40 Boys: 46.3% Girls: 53.7%. UK	Analyze the effect of CLS intervention during peripheral venous angiocatheter insertion on children’s suffering	Social Interaction	CLS (Group 1)	Before and during	Usual procedure. Standard parental accompaniment	Behavioral Distress (OSBD-R).	There was a significant reduction in distress in the CLS group (*M* = 2.83) compared to the control group (*M* = 4.63, *p* < 0.05).
Uzsen et al., 2024 (57)	120 (5 to 10 years) M = 7.56/SD = 1.72 Boys: 51.8% Girls: 48.2%. Turkey	Evaluate the effects of Buzzy and Acupressure methods on children’s pain, fear, and anxiety levels during intramuscular injection	Somato-Sensory	Buzzy or Vibration (Group 1) and Acupressure (Group 2)	Before and during	Usual procedure. Standard parental accompaniment	Fear (CFS), Pain (WB-FACES), both self-reported and observed.	Buzzy and Acupressure significantly reduced pain and fear in both observed and self-reported measures. For pain, Control (*M* = 5.58/6.26), Acupressure (*M* = 3.76/4.18), and Buzzy (*M* = 1.85/2.10). For fear, Control (*M* = 2.75/3.10), Acupressure (*M* = 1.51/1.52), and Buzzy (*M* = 1.57/1.28).
Van der Heijden et al., 2019 (70)	191 (3 to 13 years) M = 7.3/SD = 3.56 Boys: 68% Girls: 32%. Netherlands	Determine the effectiveness of music and cartoons in reducing pain and stress during emergency procedures	Screen-Based Unisensory Distractions	Cartoons on Tablet or Portable DVD (Group 1) and Listening to Music (Group 2)	During	Usual procedure. Standard parental accompaniment	Self-reported Pain (FPS-R), Observed Pain (AHTPS), Behavioral Distress (OSBD-R). Health worker and Research Observer. HR.	Observed pain showed significant differences (Control/Cartoons/Music: *M* = 3.10/2.86/2.0, *p* = 0.017), while self-reported pain did not (Control/Cartoons/Music: *M* = 3.93/3.54/2.19, *p* = 0.077), nor did distress (*M* = 1.34/1.63/1.24, *p* = 0.55) or HR (*M* = 112.87/114.2/110.07, *p* = 0.825).
Wolyniez et al., 2013 (85)	47 (3 to 16 years) M = 7.45/SD = 4.0 Boys: 70.2% Girls: 29.8%. Israel	Evaluate the effect of therapeutic clowns on stress and anxiety levels during emergency procedures	Social Interaction	Medical Clowns (Group 1)	Before and during	Usual procedure. Standard parental accompaniment	Self-reported Pain (FPS-R; VAS).	There were no significant differences in pain for FPS-R/VAS (Control: *M* = 3.3/4.5; Clowns: *M* = 1.6/4, *p* = 0.18/0.68).
Yildirim et al., 2023 (58)	150 (4 to 10 years) M = 6.5/SD = 1.63 Boys: 52.7% Girls: 47.3%. Turkey	Determine whether using VR glasses or Buzzy during IV insertion improves first attempt success and reduces pain, fear, and anxiety levels	Immersive Reality Somato-Sensory	Video with VR Glasses (Group 1) and Buzzy (Group 2)	During and after	Usual procedure. Standard parental accompaniment	Self-reported Pain (WB-FACES; CAS), and Fear (CFS), Behavioral Distress (CEAS). Biological Markers: BP, RR, HR.	No significant differences were found in self-reported pain with WB or CAS (Control/VR/Buzzy: *M* = 6.2/5.6/5.9 and 6.0/5.9/5.8, *p* > 0.20). Clinically relevant differences in fear were noted (Control/VR/Buzzy: *M* = 3.2/3.0/2.9, *p* = 0.033). No significant differences were found in distress (Control/VR/Buzzy: *M* = 20.1/18.8/18.3, *p* = 0.083), nor in HR (*p* = 0.766), RR (*p* = 0.335), or BP (*p* = 0.812).
Yilmaz et al., 2019 (59)	160 (5 to 10 years) M = 7.07/SD = 1.49 Boys: 48.8% Girls: 51.2%. Turkey	Compare the effectiveness of Buzzy, ShotBlocker, and Bubble Blowing in alleviating pain and fear during intramuscular injection	Somato-Sensory Active Command	Buzzy (Group 1), ShotBlocker (Group 2), and Bubble Blowing (Group 3)	Before and during	Usual procedure. Standard parental accompaniment	Pain (Oucher Scale), Fear (CFS), both self-reported and observed Caregiver and research observer.	Significant differences in both observed and self-reported (*p* < 0.05) for pain, Control (*M* = 6.58/6.72), Bubbles (*M* = 5.39/4.75), ShotBlocker (*M* = 4.37/4.14), and Buzzy (*M* = 3.14/3.87). For fear, Control (*M* = 2.73/2.82), Bubbles (*M* = 1.84/1.88), ShotBlocker (*M* = 1.61/1.66), and Buzzy (*M* = 1.39/1.35).
Zengin et al., 2022 (60)	224 (7 to 10 years) M = 8.49/SD = 1.17 Boys: 49.7% Girls: 50.3%. Turkey	Evaluate the effectiveness of ShotBlocker and Palm Stimulator in reducing pain associated with intramuscular injection	Somato-Sensory Active Command	ShotBlocker (Group 1) and Palm Stimulator (Group 2)	Before and during	Usual procedure. Standard parental accompaniment	Fear (CFS), Pain (VAS; FPS-R), both self-reported and observed-reported. Caregiver and research observer.	*Palm Stimulator* was the most effective in reducing self-reported and observed reported pain (*M* = 2.79/2.47), followed by ShotBlocker (*M* = 4.52/5.05), and control (*M* = 5.43/6.0, *p* < 0.01). For fear, there were no significant differences for both measures: Palm Stimulator (*M* = 2.00/1.81), ShotBlocker (*M* = 2.05/1.79), and control (*M* = 2.03/1.88, *p* = 0.98).

AHTPS, Alder Hey Triage Pain Score; BP, blood pressure; CAM, child anxiety meter; CAM-S, children's anxiety meter-state; CAPS, children anxiety and pain scales—anxiety; CAS, color analog scale; CAS-S, child anxiety statement scale; CEMS, children's emotional manifestation scale; CEAS, children's emotional appearance scale; CLS, child life specialists; CMFS, children's medical fear scale; CFI, child fear inventory; Ditto-C, Ditto combined distraction-preparation; Ditto-D, Ditto distraction; Ditto-PP, Ditto procedural preparation; FLACC, faces, legs, activity, cry and consolability scale; FPS-R, faces pain scale-revised; FSSC-R, fear survey schedule for children–revised; GDS, groninger distress scale; GFS, glasses fear scale; HR, heart rate; LTAS, likert-type anxiety scales; NRS, numeric rating scale; OSBD-R, observational scale of behavioral distress-revised; PBCL, procedural behavior checklist; PSP-VG, playstation portable video games; RR, respiratory rate; SpO_2_, oxygen saturation; VAS, visual analog scale; VSAS, venham situational anxiety scale; VAT, visual analog thermometer; WB-FACES, Wong-Baker FACES scale; VR, virtual reality.

### Risk of bias

The risk of bias was assessed using the Cochrane Collaboration's Tool (46). This tool classifies studies according to the risk of bias in six domains: random sequence generation, allocation concealment, blinding of participants and personnel, blinding of outcome assessment, and incomplete outcome data. Each study was categorized as having high, moderate, or low risk in each domain, allowing for a nuanced and accurate assessment of the methodological quality and internal validity of the trials included in the analysis. The risk of bias for each included studies was assessed by two independent reviewers (MB and IR), with conflicts resolved by a third reviewer (LC).

### Meta-analysis

A bibliometric analysis of the selected RCTs was performed to gain an in-depth overview of the study characteristics and obsolescence across all the evidence. Obsolescence was calculated by means of the Burton-Kebler (Calculates the median age of cited articles to determine the “half-life” of the literature) and Prince index (Measures the percentage of cited articles that are less than a specified 5 years).

Meta-analysis was conducted to calculate pooled effect sizes of psychosensory interventions on self-reported or observed pain, fear, and behavioral distress. Review Manager (RevMan) Web version endorsed by the Cochrane Collaboration was used, to compute the effect sizes (*Z*-value and standardized mean difference [SMD]) and design of the forest plots. Effect sizes are reported along with a 95% CI and presented both quantitatively and graphically using forest plots. In these forest plots, each trial is visually represented as a horizontal diamond shape, where the center indicates the effect size, and the end points represent the CI limits.

Random-effects models (inverse-variance method) were selected a priori, given the anticipated clinical and methodological diversity among studies. Heterogeneity was assessed using the Chi^2^ and *I*^2^ statistics. The Chi^2^ statistic is used to determine if the variation among studies is significant. A low Chi^2^ and high *p*-value indicate no significant heterogeneity, suggesting consistency across studies. A high Chi^2^ and low *p*-value indicate significant heterogeneity, suggesting variations greater than expected by chance. On the other hand, the *I*^2^ statistic measures is used to determine the proportion of total variation due to heterogeneity. *I*^2^ providing a percentage that indicates the degree of inconsistency in the results, and *I*^2^ value of 25% indicates low heterogeneity, 50% moderate, and 75% high heterogeneity.

Subgroup analyses were pre-registered in PROSPERO to explore potential differences by (a) procedure type, (b) age range, and (c) adult intervention (type and if was performed by parental or professional). Quantitative subgroup analysis was feasible only by intervention modality, as mostly included studies involved venipuncture or intravenous procedures, and only one study specified exclusive parental implementation. No meta-regression was conducted, given the limited number of studies per subgroup and the diversity of designs.

Accordingly, effect sizes were calculated for seven intervention subtypes, identified and analyzed based on the type of stimulus involved in each psychosensory strategy: (1) Somato-Sensory includes interventions providing direct tactile or kinesthetic stimulation, such as vibratory (Buzzy) or cold devices (Cold Stray, Ice gel) and tactile stimulators (e.g., shotblocker). (2) Immersive Reality, which uses Virtual reality (VR) devices to engage children in simulated experiences, either through passive observation or active interaction. (3) Screen-based, uses audio-visual distractions screens, such as cartoons video and video games. (4) Toy interaction use toys to capture the child's attention during the procedure, with or without handling. (5) Social interaction includes dynamic activities with others (e.g., medical clowns, robots, caregivers or child life specialists (CLS). (6) Active command, involve simple commands to cope with the situation and require the child's active participation, such as squeezing a stress ball, whistling, or blowing soap bubbles. (7) Unisensory distractions involve simple cognitive or visual interventions, like viewing cards, vein imaging device (VID) or listening to music, without combining multiple sensory stimuli.

Sensitivity analyses were not performed, as the primary objective of this review was to estimate the overall effectiveness of psychosensory interventions across heterogeneous designs. Given the diversity of interventions and outcome measures, excluding studies based on methodological quality or sample size would have substantially reduced statistical power.

## Results

### Article selection

A total of 1,796 articles were identified by the searches. After removing 269 duplicates, 1,527 titles and abstracts were screened, revealing 155 abstracts meeting the inclusion criteria for full-text screening. After full-text screening, 45 articles were selected for final inclusion, one of which was identified through backward snowballing after full-text screening. The main reasons for exclusion were: (1) irrelevant sample, as some studies included adults or focused on age groups not related to the review, such as neonates; (2) not pertinent and setting intervention with studies not applied in PED; (3) unrelated intervention, with some studies addressing pharmacological approaches instead of psychological or sensory interventions; (4) inappropriate type of procedure, excluding studies that did not focus on IMPs; (5) non-experimental or quasi-experimental study design; and (6) absence of relevant outcomes, where studies did not measure outcomes such as pain, fear, or distress during the procedure (see Figure 1).

The total sample size across the 45 articles was *N* = 6,480 children (3,087 females and 3,393 males). The mean age of participants was 7.34 with a of SD 3.26. The mean age of articles was 3.76 years, with a SD of 4.57, 95% CI [3.24, 5.91]. The most recent article was 4 months old, with the oldest being 20 years old. The obsolescence of the RCTs, considering a Burton-Kebler index and Prince index, showed a median of 2 years, with 84.44% of the RCTs being less than 5 years old. The 45 studies were conducted in 13 countries, mainly in Turkey (*n* = 19; 42.2%), Canada (*n* = 9; 20.0%), and the United States (*n* = 4; 8.9%). The remaining studies came from 10 other countries across Europe, Asia, and the Middle East (see Table 1).

The following sections present the outcomes for pain, fear, and distress, as reported by either children or observers, across all the interventions as well as grouped by these intervention categories. Results are presented in order of effectiveness of the intervention category. Due the limited findings for biomarkers, the results for biomarkers could not be organized by intervention category, hence these findings are organized according to the assessed biomarker instead.

### Pain

Thirty-seven studies evaluated the effectiveness in reducing pain, demonstrating significant reductions in both self-reported and observer-reported pain. For self-reported pain, the overall effect test yielded a *Z* value of 7.66 (*p* < 0.01), with a total SMD of −0.94 (95% CI: −1.19, −0.70). Subgroup analysis revealed substantial heterogeneity, evidenced by Chi^2^454.17 (df = 17, *p* < 0.01) and an *I*^2^ of 96.3%. For observer-reported pain the overall effect yielded a *Z* value of 6.27 (*p* < 0.01) with an SMD of −1.52 (95% CI: −2.05, −0.99). The substantial heterogeneity was evidenced by Chi^2^264.40 (df = 11, *p* < 0.01), reflecting an *I*^2^ 95.8%. Below is a summary for the effectiveness of the intervention category, ordered by the efficacy of the interventions (SMD, *Z* and *p*-values are in Table 2).

**Table 2 (Part A):** Summary of *Z*-scores, *p*-values, and SMD of pain, fear and distress during the procedure.

Intervention	Self-Reported Pain			Self-Reported Fear			Observed Pain			Observed Fear			Observed Distress		
	Z	p	SMD + IC	Z	p	SMD + IC	Z	p	SMD + IC	Z	p	SMD + IC	Z	p	SMD + IC
**Active Command**	**6.98**	**<0.01**	**−0.94 [−1.21, −0.68]**	**3.86**	**<0.01**	**−1.31 [−1.98, −0.65]**	**3.88**	**<0.01**	**−1.22 [−1.84, −0.60]**	**2.62**	**<0.01**	**−1.30 [−2.27, −0.33]**	N. E	N. E	N. E
Blowing Soap Bubbles	6.87	<0.01	−1.06 [−1.37, −0.76]	9.41	<0.01	−1.57 [−1.90, −1.24]	1.49	0.14	−1.05 [−2.44, 0.34]	4.41	<0.01	−1.73 [−2.50, −0.96]	2.69	<0.01	−0.92 [−1.59, −0.25]
Palm Stimulator	6.84	<0.01	−1.00 [−1.29, −0.71]	0.10	0.92	−0.02 [−0.40, 0.36]	5.90	<0.01	−1.26 [−1.68, −0.84]	0.25	0.80	−0.05 [−0.43, 0.33]	N. E	N. E	N. E
Stress Ball	4.56	<0.01	−1.15 [−1.64, −0.66]	4.22	<0.01	−1.05 [−1.54, −0.56]	5.83	<0.01	−1.56 [−2.08, −1.03]	6.22	<0.01	−1.70 [−2.24, −1.17]	N. E	N. E	N. E
Whistling Breathing	1.99	0.05	−0.45 [−0.89, −0.01]	N. E	N. E	N. E	N. E	N. E	N. E	N. E	N. E	N. E	N. E	N. E	N. E
**Immersive Reality:**															
Video Game with VR	**0.03**	**0.98**	**−0.00 [−0.27, 0.27]**	**0.95**	**0.34**	**−0.13 [−0.40, 0.14]**	N. E	N. E	N. E	N. E	N. E	N. E	3.02	<**0.01**	**−0.80 [−1.32, −0.28]**
**Immersive Reality:**	**4.36**	<**0.01**	**−0.93 [−1.34, −0.51]**	2.69	<0.01	**−1.23 [−2.13, −0.34]**	N. E	N. E	N. E	N. E	N. E	N. E	3.51	<**0.01**	**−0.75 [−1.17, −0.33]**
Video VR Glasses	3.89	<0.01	−0.91 [−1.36, −0.45]	2.49	0.01	−1.39 [−2.48, −0.29]	1.42	0.16	−1.38 [−3.29, 0.53]	1.25	0.21	−2.42 [−6.21, 1.37]	4.66	<0.01	−0.84 [−1.19, −0.49]
Video VR Glasses + VID	3.31	<0.01	−0.86 [−1.36, −0.35]	2.17	0.03	−0.49 [−0.94, −0.05]	N. E	N. E	N. E	N. E	N. E	N. E	N. E	N. E	N. E
**Screen-Based: Cartoons**	**1.62**	**0.11**	**−0.95 [−2.09, 0.20]**	**2.10**	**0.04**	**−2.31 [−4.48, −0.15]**	**1.86**	<0.01	**−2.66 [−4.58, −0.75]**	**10.68**	<**0.01**	**−3.35 [−3.96, −2.73]**	**1.24**	**0.21**	**−0.47 [−1.21, 0.27]**
**Screen-Based: Ditto**	**1.33**	**0.18**	**−0.25 [−0.61, 0.12]**	N. E	N. E	N. E	**4.39**	<**0.01**	**−0.94 [−1.37, −0.52]**	N. E	N. E	N. E	**2.83**	<**0.01**	**−0.53 [−0.90, −0.16]**
Ditto C	0.94	0.34	−0.30 [−0.94, 0.33]	N. E	N. E	N. E	3.87	<0.01	−1.40 [−2.11, −0.69]	N. E	N. E	N. E	1.32	0.19	−0.43 [−1.06, 0.21]
Ditto-D	1.29	0.20	−0.41 [−1.04, 0.21]	N. E	N. E	N. E	2.07	0.04	−0.67 [−1.31, −0.04]	N. E	N. E	N. E	2.16	0.03	−0.71 [−1.35, −0.06]
Ditto-P	0.06	0.95	−0.02 [−0.65, 0.61]	N. E	N. E	N. E	2.46	0.01	−0.83 [−1.48, −0.17]	N. E	N. E	N. E	1.43	0.15	−0.46 [−1.10, 0.17]
**Informational Video**	**21.54**	<**0.01**	**−4.72 [−5.15, −4.29]**	**21.24**	<**0.01**	**−4.49 [−4.90, −4.07]**	**21.36**	<**0.01**	**−4.58 [−5.00, −4.16]**	**20.92**	<**0.01**	**−4.26 [−4.66, −3.86]**	N. E	N. E	N. E
**Screen-Based: Video Games**	1.73	0.08	−0.56 [−1.19, 0.08]	N. E	N. E	N. E	1.98	0.05	−0.64 [−1.28, −0.01]	N. E	N. E	N. E	2.76	<0.01	−0.92 [−1.58, −0.27]
**Screen-Based: VR Ceiling**	N. E	N. E	N. E	N. E	N. E	N. E	N. E	N. E	N. E	N. E	N. E	N. E	1.08	0.28	−0.23 [−0.65, 0.19]
**Simple Cognitive**	**2.90**	<**0.01**	**−1.50 [−2.51, −0.48]**	N. E	N. E	N. E	N. E	N. E	N. E	N. E	N. E	N. E	N. E	N. E	N. E
Distraction Cards	5.40	<0.01	−0.72 [−0.98, −0.46]	N. E	N. E	N. E	N. E	N. E	N. E	N. E	N. E	N. E	N. E	N. E	N. E
Listening to Music	2.05	0.04	−1.61 [−3.14, −0.07]	6.47	<0.01	−1.83 [−2.39, −1.28]	N. E	N. E	N. E	N. E	N. E	N. E	0.31	0.76	**−0.06 [−0.41, 0.30]**
**Simple Visual:**	**0.00**	**1.00**	**0.00 [−0.44, 0.44]**	N. E	N. E	N. E	N. E	N. E	N. E	N. E	N. E	N. E	N. E	N. E	N. E
Cartoon-Clothes	2.19	0.03	−0.74 [−1.40, −0.08]	2.59	0.01	−0.88 [−1.55, −0.21]	N. E	N. E	N. E	N. E	N. E	N. E	2.19	0.03	**−0.74 [−1.40, −0.08]**
Vein Imaging Device	0.00	1.00	0.00 [−0.31, 0.31]	0.77	0.44	−0.17 [−0.61, 0.27]	N. E	N. E	N. E	N. E	N. E	N. E	N. E	N. E	N. E
**Social Interaction:**															
Child Life Specialists	N. E	N. E	N. E	N. E	N. E	N. E	N. E	N. E	N. E	N. E	N. E	N. E	0.72	0.47	−0.12 [−0.46, 0.21]
Finger Puppets	N. E	N. E	N. E	N. E	N. E	N. E	N. E	N. E	N. E	N. E	N. E	N. E	7.38	<0.01	−2.06 [−2.60, −1.51]
Medical Clowns	1.16	0.25	−0.61 [−1.63, 0.42]	2.82	<0.01	**−0.95 [−1.60, −0.29]**	N. E	N. E	N. E	N. E	N. E	N. E	N. E	N. E	N.E
Parental Distraction	0.81	0.42	−0.25 [−0.85, 0.35]	1.02	0.31	−0.32 [−0.92, 0.29]	N. E	N. E	N. E	N. E	N. E	N. E	1.51	0.13	−0.25 [−0.85, 0.35]
Robot Coach	2.92	0.003	−0.67 [−1.12, −0.22]	N. E	N. E	N. E	N. E	N. E	N. E	N. E	N. E	N. E	1.65	0.10	−0.37 [−0.81, 0.07]
**Somatosensory:**															
Buzzy or Vibration	4.68	<0.01	−1.14[−1.62, −0.66]	5.01	<0.01	−2.30 [−3.20, −1.40]	4.97	<0.01	−2.15 [−2.99, −1.30]	4.90	<0.01	−2.69 [−3.77, −1.61]	1.46	0.14	−0.57 [−1.34, 0.20]
Cold Spray	2.54	0.01	−2.35 [−4.17, −0.53]	13.29	<0.01	−3.11 [−3.57, −2.65]	2.42	0.02	−2.01 [−3.64, −0.38]	6.55	<0.01	−2.66 [−3.46, −1.87]	N. E	N. E	N. E
Ice Gel	1.61	0.11	−0.41 [−0.90, 0.09]	1.99	0.05	−0.50 [−1.00, −0.01]	1.86	0.06	−0.47 [−0.97, 0.02]	1.80	0.07	−0.46 [−0.95, 0.04]	N. E	N. E	N. E
Shotblocker	4.15	<0.01	−0.62 [−0.92, −0.33]	2.32	0.02	−1.17 [−2.16, −0.18]	2.64	<0.01	−0.91 [−1.58, −0.23]	2.40	0.02	−1.18 [−2.14, −0.21]	N. E	N. E	N. E
Tactile Manual	1.73	0.08	−1.21 [−2.58, 0.16]	1.34	0.18	−0.97 [−2.38, 0.45]	1.68	0.09	−0.48 [−1.04, 0.08]	1.29	0.20	−0.88 [−2.21, 0.46]	N. E	N. E	N. E
**Toy Interaction:**	**16.52**	<**0.01**	**−1.76 [−1.97, −1.55]**	**2.90**	<**0.01**	**−1.35 [−2.27, −0.44]**	**6.27**	<**0.01**	**−3.35 [−4.39, −2.30]**	**2.14**	**0.03**	**−1.89 [−3.62, −0.16]**	N. E	N. E	N. E
Musical Bracelet	4.63	<0.01	−2.04 [−2.60, −1.48]	8.24	<0.01	−1.58 [−1.95, −1.20]	11.91	<0.01	−2.82 [−3.29, −2.36]	10.48	<0.01	−2.24 [−2.66, −1.82]	N. E	N. E	N. E
Kaleidoscope	6.33	<0.01	−1.78 [−2.33, −1.23]	6.21	<0.01	−1.73 [−2.28, −1.18]	N. E	N. E	N. E	N. E	N. E	N. E	N. E	N. E	N. E
Rotating Tactile	13.31	<0.01	−1.90 [−2.18, −1.62]	10.31	<0.01	−2.18 [−2.60, −1.77]	13.60	<0.01	−3.89 [−4.45, −3.33]	12.79	<0.01	−3.31 [−3.81, −2.80]	N. E	N. E	N. E
Musical and Dancing	N. E	N. E	N. E	0.37	0.71	0.10 [−0.41, 0.60]	N. E	N. E	N. E	0.46	0.65	−0.12 [−0.62, 0.39]	N. E	N. E	N. E

CI, confidence interval; Ditto-C, Ditto combined distraction-preparation; Ditto-D, Ditto distraction; Ditto-PP, Ditto procedural preparation; N.E., not evaluated in the study; PSP, play station portable; SMD, standardized mean differences; VID, vein imaging device; VR, virtual reality; Z, *Z*-scores; *p*, *p*-values.

Bold values indicate statistically significant effects at *p* < 0.05. SMD + CI refers to the standardized mean difference with 95% confidence interval.

**Table 2 (Part B):** Summary of *Z*-scores, *p*-values, and SMD of biological markers during the procedure.

Intervention	Biological Marker	Z-Value	*P*-Value	SMD + IC
Somatosensory: Buzzy or Vibration	Respiratory Frequency	0,15	0,88	0.03 [−0.36, 0.42]
	Diastolic Blood Pressure	−0,72	0,47	−0.15 [−0.54, 0.25]
	Systolic Blood Pressure	0,28	0,78	0.06 [−0.34, 0.45]
	Heart Rate	−0,46	0,65	−0.09 [−0.49, 0.30]
Screen-Based: Cartoons	Heart Rate	0.73	0.47	0.16 [−0.27, 0.58]
Social Interaction: Robot Coach	Heart Rate	0.18	0.86	0.04 [−0.38, 0.46]
Simple Cognitive: Listening to Music	Heart Rate	−0,27	0,79	−0.08 [−0.69, 0.52]
Social Interaction: Medical Clowns	Cortisol	−0,46	0,64	−0.13 [−0.67, 0.41]
Toy Interaction: Musical and Dancing	Heart Rate	1,57	0,12	0.41 [−0.10, 0.92]
	Oxygen Saturation (SpO2)	−0,79	0,43	−0.20 [−0.71, 0.30]
Immersive Reality: Video with VR Glasses	Respiratory Frequency	−1,16	0,24	−0.23 [−0.63, 0.16]
	Diastolic Blood Pressure	0,73	0,46	0.15 [−0.25, 0.54]
	Systolic Blood Pressure	−0,38	0,7	−0.08 [−0.47, 0.32]
	Heart Rate	−0,61	0,54	−0.12 [−0.51, 0.27]

CI, confidence interval; SMD, standardized mean differences; VR, virtual reality; *Z*, *Z*-scores; *p*, *p*-values.

**Table 2 (Part C):** Summary of *Z*-scores, *p*-values, and SMD of biological markers after the procedure.

Intervention	Biological Marker	Z Value	P Value	SMD + IC
Somatosensory: Buzzy or Vibration	Respiratory Frequency	−0,93	0,35	−0.19 [−0.58, 0.21]
	Heart Rate	−1,04	0,3	−0.21 [−0.60, 0.19]
Screen-Based: Cartoons	Heart Rate	−9,21	<0.00001	−1.11 [−1.35, −0.87]
	Oxygen Saturation (SpO2)	0,34	0,74	0.04 [−0.18, 0.26]
	Systolic Blood Pressure	−6,56	<0.00001	−0.76 [−0.99, −0.54]
	Diastolic Blood Pressure	−5,3	<0.00001	−0.61 [−0.83, −0.38]
	Heart Rate	0,27	0,79	0.05 [−0.32, 0.43]
Simple Cognitive: Listening to Music	Heart Rate	−0,67	0,5	−0.12 [−0.48, 0.24]
Toy Interaction: Musical and Dancing	Heart Rate	2,91	0,004	0.78 [0.25, 1.31]
	Oxygen Saturation (SpO2)	−1,88	0,06	−0.49 [−1.01, 0.02]
Screen-Based: Informational Video	Heart Rate	−8,35	<0.00001	−0.99 [−1.23, −0.76]
	Oxygen Saturation (SpO2)	0,54	0,59	0.06 [−0.16, 0.28]
	Systolic Blood Pressure	−7,17	<0.00001	−0.84 [−1.07, −0.61]
	Diastolic Blood Pressure	−5,66	<0.00001	−0.65 [−0.88, −0.43]
Immersive Reality: Video VR Glasses	Respiratory Frequency	−1,16	0,25	−0.23 [−0.63, 0.16]
	Heart Rate	6,76	<0.00001	1.55 [1.10, 2.00]

CI, confidence interval; SMD, standardized mean differences; VR, virtual reality; *Z*, *Z*-scores; *p*, *p*-values.

**Table 3:** Summary measures and application timing.

Intervencion	Sample	Measure Self-report			Observed Behavior			Biological markers		
		Before	During	After	Before	During	After	Before	During	After
**Active Comand:**										
Palm Stimulator	School Age (60)	Fear: CFS		Pain: VAS, FPS-R	Fear: CFS	Pain: FPS-R				
Blowing Soap Bubbles	Preschool (87) School Age (59, 80, 87)	Pain: WB-FACES (80). Fear: CFS (59, 80)	Pain: Oucher (59). Fear: CFS (59, 87)	Pain: WB-FACES. Fear: CFS (80)	Pain: WB-FACES (80), Fear: CFS (59, 80)	Pain: Oucher (59). Fear: CFS (59). Distress: FLACC (87)	Pain: WB-FACES. Fear: CFS (80)			
Shotblocker	School Age (49, 51, 55, 56, 59, 60)	Pain: WB-FACES (49, 51), VAS, FPS-R (55, 56). Fear: CFS (49, 51, 59, 60)	Pain: Oucher. Fear: CFS (59).	Pain: WB-FACES (49, 51), VAS, FPS-R (55, 56, 60). Fear: CFS (49, 51)	Pain: WB-FACES (49, 51). Fear: CFS (49, 51 59, 60)	Pain: WB-FACES, Oucher (59) Fear: CFS (59), FPS-R (60)	Pain: WB-FACES. Fear: CFS (49, 51)			
Stress Ball	School Age (80)	Pain: WB-FACES. Fear: CFS		Pain: WB-FACES. Fear: CFS	Pain: WB-FACES. Fear: CFS		Pain: WB-FACES. Fear: CFS			
Whistling Breathing	Preschool & School Age (53)	Pain WB-FACES	Pain WB-FACES	Fear CMFS						
**Immersive reality:**										
Video Game with Virtual Reality	Preschool (79), School Age (68, 78, 79), Teenager (78, 79)	Pain: VAS (68), VNRS (78), FPS-R (79). Fear: VAS (68), CFS (78), LTAS (79)	Pain: VNRS. Fear: CFS (78)	Pain: VAS (68) VNRS (78), FPS-R (79). Fear: VAS (68), CFS (78), LTAS (79)	Pain: FPS-R. Fear: LTAS (79). Distress: PBCL (78)	Distress PBCL (78)	Pain: FPS-R. Fear: LTAS (79)			
Video with Virtual Reality Glasses	Preschool (58, 74, 75), School Age (58, 65, 72, 74–77, 88), Teenager (77)	Pain: FPS-R (75, 77), WB-FACES (72). Fear: CFS (65), VAT (75), CAM-S, CMFS (72)	Pain: FPS-R (75), WB-FACES (76). Fear: CFS (74, 76), VAT (75)	Pain: WB-FACES (58, 65, 72, 74), CAS (58, 74), FPS-R (77). Fear: CFS (58, 65), CAM-S, CMFS (72)	Fear: CFS (65). Distress: CEMS (74), CEAS (58)	Pain: WB-FACES (76). Fear: CFS (65, 76), Distress: CEMS (74, 88)	Distress CEMS (88)	BP, HR, RR (58)	BP, HR, RR (58)	BP, HR, RR (58)
Video with Virtual Reality Glasses + VID	Preschool & School Age (74)			Pain: WB-FACES, CAS. Fear: CFS	Distress CEMS		Distress CEMS			
**Screen Based:**										
Ditto D-PP-C	Preschool & School Age (64)	Pain WB-FACES	Pain WB-FACES	Pain WB-FACES	Pain: VAS. Distress: FLACC	Pain: VAS. Distress: FLACC	Pain: VAS. Distress: FLACC			
Informational Video	School Age (62)	Pain: WB-FACES. Fear: CFS		Pain: WB-FACES. Fear: CFS	Pain: WB-FACES. Fear: CFS		Pain WB-FACES, Fear CFS	SpO2, BP, HR		SpO2, BP, HR
PSP Video Games	Preschool & School Age (64)	Pain WB-FACES	Pain WB-FACES	Pain WB-FACES	Pain: VAS. Distress: FLACC	Pain: VAS. Distress: FLACC	Pain: VAS. Distress: FLACC			
VR Ceiling Screen	Infant/Preschool (63)				Distress FLACC, Pain and Fear VAS					
Cartoons	Preschool (67, 69, 70), School Age (62, 65–70), Teenager (67)	Pain: FPS-R (66, 67 70), VAS (68), WB-FACES (62). Fear: CFS (62, 65), VAS (68)	Pain: WB-FACES (69), FPS-R (66, 67)	Pain: FPS-R (67, 70), WB-FACES (62, 65), VAS (68). Fear: CFS (62, 65), VAS (68)	Pain WB-FACES (62). Fear: CFS (65, 62). Distress: OSBD-R (66, 70), GDS (69)	Pain: WB-FACES (65) AHTPS (70). Fear: CFS (65), Distress: OSBD-R (66, 70), GDS (69)	Pain: WB-FACES, Fear: CFS (62). Distress: OSBD-R (66, 70),	SpO2, BP (62), HR (62, 69, 70)	HR (66, 69)	SpO2, BP (62), HR (62, 69, 70)
**Simple Cognitive:**										
Distraction Cards	School Age (56)	Pain: VAS, FPS-R		Pain: VAS, FPS-R						
Listening to Music	Preschool (61, 70, 73), School Age (61, 70, 72, 73)	Pain: FPS-R (73, 70), WB-FACES. Fear: CAM-S, CMFS (72)	Pain: FPS-R (73), Oucher (61)	Pain: FPS-R (70, 73), WB-FACES. Fear: CAM-S, CMFS (72).	Distress: OSBD-R (73)	Pain: AHTPS (70). Distress: OSBD-R (73)	Distress: OSBD-R (73)	HR (70)	HR (73)	HR (70)
**Simple Visual:**										
Vein Imaging Device	Preschool, School Age &Teenager (74)		Fear CFS	Pain: WB-FACES, CAS	Distress CEMS	Distress CEMS				
Cartoon-Clothes	Preschool & School Age (87)		Fear CFS			Distress FLACC				
**Social Interaction:**										
Child Life Specialists	Infant/Preschool, School Age & Teenager (90)					Distress OSBD-R				
Finger Puppets	Preschool & School Age (89)				Distress CEMS	Distress CEMS				
Medical Clowns	Infant/Preschool (83–85), School Age (83–85), Teenager (84, 85)	Pain: WB-FACES. Fear: CAPS (83)	Pain: WB-FACES (83), VAS, FPS-R (84, 85). Fear: CAPS (83)		Pain: NRS (83)	Pain: NRS (83)			Plasma Cortisol (84)	
Parental Positioning and Distraction	Preschool & School Age (82)		Pain WB-FACES, Fear GFS		Fear: GFS. Distress: PBCL	Distress: PBCL	Fear: GFS. Distres:s PBCL			
Robot Coach	School Age (81)	Pain FPS-R	Pain FPS-R		Distress: OSBD-R	Distress: OSBD-R	Distress: OSBD-R		HR	
**Somatosensory:**										
Buzzy or Vibration	Preschool (52, 53, 57, 58, 91), School Age (47, 51, 52, 54–59), Teenager (47, 52)	Pain: CAPS (47), WB-FACES (51, 53), FPS-R (52, 55, 56), VAS (55, 56). Fear: CFS (51, 57, 59)	Pain: FPS-R (47, 52), WB-FACES (53, 54, 57), Oucher (59) Fear: CFS (57, 59), CFI (54)	Pain: WB-FACES (51, 58), VAS, FPS-R (55, 56). CAS (58). Fear: CMFS (53), CFI (51), CFS (58)	Pain: CAPS (47), WB-FACES (51). Fear: CFS (51, 57, 59), Distress: CEAS (58)	Pain: FPS-R (47). WB-FACES (54, 57), Oucher (59). Fear: CFS (57, 59), CFI (54) Distress: OSBD-R (47, 52), CHEOPS (91), FLACC (52)	Pain: WB-FACES (51). Fear: CFS (51)	BP, HR, RR (58)	BP, HR, RR (58)	BP, HR, RR (58)
Cold Spray	School Age (48, 50, 54), Teenager (48)	Pain: VAS. Fear: CFS (48)	Pain: VAS (48, 50), WB-FACES (54). Fear: CFS (48), CFI (54)		Pain: VAS. Fear: CFS (48)	Pain: VAS (48), WB-FACES (54). Fear: CFS (48), CFI (54)				
Ice Gel	School Age & Teenager (48)	Pain: VAS. Fear: CFS	Pain: VAS. Fear: CFS		Pain: VAS. Fear: CFS	Pain: VAS. Fear: CFS				
Manual Tactil	Preschool (61, 57), School Age (49, 57, 61)	Pain: WB-FACES (49). Fear: CFS (49, 57)	Pain: WB-FACES (57), Oucher (61). Fear: CFS (57)	Pain: WB-FACES. Fear: CFS (49)	Pain: WB-FACES Fear: CFS (49, 57)	Pain: VAS (50), WB-FACES. Fear: CFS (57)	Pain: WB-FACES. Fear: CFS (49)			
**Toy Interaction:**										
Kaleidoscope	School Age (72)	Pain: WB-FACES. Fear: CAM-S, CMFS		Pain: WB-FACES. Fear: CAM-S, CMFS						
Musical and Dancing	Preschool & School Age (86)	Fear CFS	Fear CFS	Fear CFS	Fear CFS	Fear CFS	Fear CFS	SpO2, HR	SpO2, HR	SpO2, HR
Musical Bracelet	School Age (71)	Pain: WB-FACES, VAS. Fear CFS	Pain: WB-FACES, VAS. Fear CFS		Pain: WB-FACES. Fear: CFS	Pain: WB-FACES. Fear: CFS				
Rotating Tactile	School Age (71)	Pain: WB-FACES, VAS. Fear CFS	Pain: WB-FACES, VAS. Fear CFS		Pain: WB-FACES. Fear: CFS	Pain: WB-FACES. Fear: CFS				

AHTPS, Alder Hey Triage Pain Score; BP, blood pressure; CAM, child anxiety meter; CAM-S, children's anxiety meter-state; CAPS, children anxiety and pain scales—anxiety; CAS, color analog scale; CAS-S, child anxiety statement scale; CEMS, children's emotional manifestation scale; CEAS, children's emotional appearance scale; CLS, child life specialists; CMFS, children's medical fear scale; CFI, child fear inventory; Ditto-C, Ditto combined distraction-preparation; Ditto-D, Ditto distraction; Ditto-PP, Ditto procedural preparation; FLACC, faces, legs, activity, cry and consolability scale; FPS-R, faces pain scale-revised; FSSC-R, fear survey schedule for children–revised; GDS, groninger distress scale; GFS, glasses fear scale; HR, heart rate; LTAS, likert-type anxiety scales; NRS, numeric rating scale; OSBD-R, observational scale of behavioral distress-revised; PBCL, procedural behavior checklist; PSP-VG, playstation portable video games; RR, respiratory rate; SpO_2_, oxygen saturation; VAS, visual analog scale; VSAS, venham situational anxiety scale; VAT, visual analog thermometer; WB-FACES, Wong-Baker FACES scale; VR, virtual reality.

Somato-Sensory (*N* = 15 studies): Buzzy or vibration, cold spray, and Shotblocker significantly reduced pain both types of reports (47–61). Manual Tactile significantly reduced observer-reported pain (57), while it only approached significance for self-reported pain (57, 61). Ice gel approached significance only for observer-reported pain (48), while it did not reach statistical significance for self-reported pain (48).

Screen-Based (*N* = 9 studies): Informational videos were effective in reducing self-reported and observer-reported pain (62). For observer-reported pain, Ditto device in its 3 versions, [Distraction (D), Preparation (PP), and both Combined (C)], ceiling-mounted VR (63, 64), and cartoons screens showed a significant effect (65–70).

Toy-Interaction (*N* = 2 studies): Rotating tactile, musical bracelet, and kaleidoscope were effective in reducing pain for both self-reported and observer-reported measures (71, 72).

Unisensory-Distractions (*N* = 5 studies): For self-reported pain, viewing cards and listening to music were effective in reducing pain (61, 70, 72, 73), while VID did not reach statistical significance (74).

Immersive Reality (*N* = 10 studies): VR glasses showed significant results for self-reported pain but did not reach significance for observer-reported pain (58, 65, 72, 74–77). VR video games were not significant in reducing self-reported pain but approached significance in observer-reported pain (68, 78, 79).

Active-Command (*N* = 4 studies): Squeezing a stress ball and the palm stimulator were significantly effective in reducing self-reported and observer-reported pain (60, 80). Blowing soap bubbles was effective in reducing self-reported pain but did not reach significance in observer-reported pain (58, 79). Whistling approached significance for self-reported pain (53).

Social-Interaction (*N* = 5 studies): Robot were significantly effective in reducing self-reported pain (81), while neither parental distraction (82) nor medical clowns (83–85) reached statistical significance in the same measure.

### Fear

Twenty-six studies evaluated the impact of interventions on their effectiveness of reducing fear. The analysis revealed a significant reduction in self-reported fear, with an overall effect test of *Z* = 6.18 (*p* < 0.01) and a total SMD of −1.30 (95% CI: −1.71, −0.89). Subgroup analysis indicated substantial variability, as reflected in the Chi^2^ = 392.44 (df = 14, *p* < 0.01) and an *I*^2^ 96%. Similarly, for observer-reported fear (evaluated in thirteen studies) a significant effect was demonstrated, with an overall effect of *Z* = 5.28 (*p* < 0.01) and an SMD of −1.77 (95% CI: −2.43, −0.16). The subgroup differences highlighted significant heterogeneity, with Chi^2^ = 166.02 (df = 9, *p* < 0.01) and an *I*^2^ 94.6%.

Somato-Sensory (*N* = 9 studies): Cold Spray, Buzzy, and Shotblocker were significantly effective in reducing both self-reported and observer-reported fear (48, 51, 53, 54, 58, 59). Ice Gel approached significance for both types of reports (48), while Manual Tactile did not reach significance in either of them (49, 57, 60).

Toy-Interaction (*N* = 3 studies): Rotating tactile, musical bracelet, and viewing a kaleidoscope were significantly effective in reducing both self-reported and observer-reported fear (71, 72). In contrast, musical and dancing toys (MDT) did not reach significance for either type of report (86).

Unisensory-Distractions (*N* = 3 studies): Listening to music was effective in reducing self-reported fear (72). Cartoon-patterned clothes demonstrated significant results for reducing self-reported fear (87), while the VID did not reach significance for either type of report (74).

Immersive-Reality (*N* = 10 studies): VR headset videos significantly reduced self-reported fear, but did not reach significance in observer-reported fear (58, 65, 72, 74–77). VR video games (68, 78, 79) showed no significant reduction in self-reported fear.

Active-Command (*N* = 5 studies): Blowing soap bubbles and squeezing a stress ball significantly reduced both self-reported and observer-reported fear (59, 80, 87). However, whistling was only effective in reducing self-reported fear (53), and Palm Stimulator was not significant in any measurement (60).

Screen-Based (*N* = 4 studies): Informational videos and cartoons were significantly effective in reducing both self-reported fear and observer-reported (62, 65, 68) and ceiling-mounted VR screens for observer-reported (63).

### Distress

The studies (*N* = 17) evaluated the effectiveness in reducing observer-reported distress in children during IMPs in PEDs and included combined results from parental, healthcare staff, and researcher perceptions. The pooled effects analysis showed a significant effect for observer-reported distress reduction (*Z* = 4.38, −0.57 [−0.83, −0.32] *p* < 0.01). The differences between subgroups were also significant (Chi^2^ = 59.84, df = 13, *p* < 0.01; *I*^2^ = 78.3%), suggesting variability in the efficacy of the different categories of intervention.

Active-Command (*N* = 1 study): Blowing soap bubbles significantly reduced distress level (87).

Immersive Reality (*N* = 5 studies): VR video games and VR goggles were effective in reducing distress (58, 74, 75, 78, 88).

Screen-Based (*N* = 4 studies): The Ditto device (64) (D-PP-C) and video games (64) showed a significant effect in reducing distress, while cartoons and ceiling-mounted VR screens did not reach significance (63, 64, 69, 70).

Social Interaction (*N* = 4 studies): Finger puppet significantly reduced observer-reported distress (89). Robot, caregivers and CLS support did not reach significance (81, 82, 90).

Unisensory-Distractions (*N* = 4 studies): Cartoon-patterned clothes (colorful animated characters), demonstrated modest but significant results (87). Listening to music was not effective in reducing distress (70, 73), and VID showed a trend toward significance (74).

Somato-Sensory (*N* = 4 studies): Buzzy were not significant in reducing distress (47, 52, 58, 91).

### Biological markers

Only seven studies evaluated biological markers during the procedure (58, 66, 69, 73, 78, 81, 86), while five studies assessed them post-procedure (58, 62, 69, 70, 86).

For Heart Rate (HR), informational videos significantly reduced post-procedural HR (62), while VR glasses and MDT significantly increased heart rate post-procedure (86). Buzzy showed no significant effect on post-procedural HR (58). During the procedure, no significant changes were observed for HR in most of the evaluated interventions (58, 62, 66, 70, 73, 81, 86). Only one study reported a significant reduction of HR (cartoon intervention, *p* < 0.01) (69) but without the mean and SD per group.

For post-procedural blood pressure, informational videos and cartoons significantly reduced both systolic and diastolic pressure (62). During the procedure, no significant effects were observed for any interventions (58). For SpO2 (Oxygen Saturation), MDT approached significance post-procedure (86), but not during the procedure. Video with VR glasses showed no significant effects during/post-procedure (58). No intervention impacted plasma cortisol levels during the procedure (84) or respiratory frequency during/post-procedure (58).

### Risk of bias assessment

**Figure 2:**
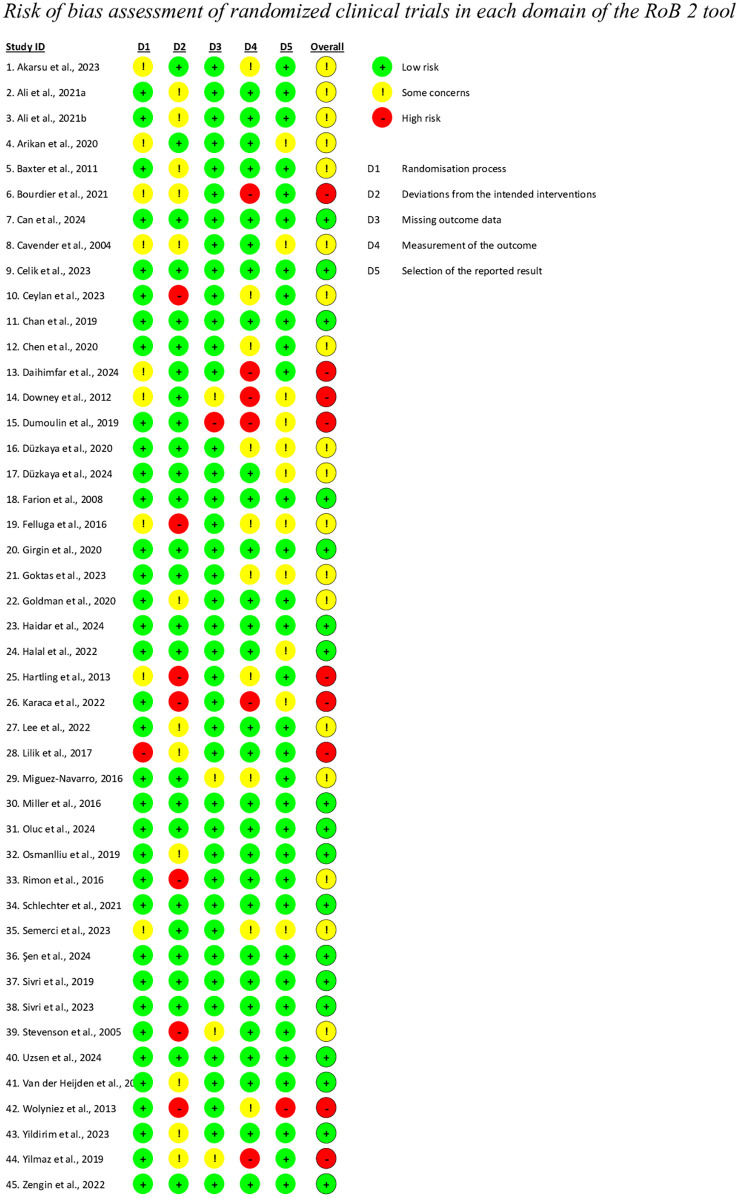
Risk of bias assessment of randomized clinical trials in each domain of the RoB-2 tool.

The risk of bias was assessed using the RoB-2 tool for RCTs, through which 40% of studies were classified as low risk, 40% as having some concerns, and 20% as high risk (see Figure 2). The risk levels with respect to the five domains were as follows: (1) the randomization process showed low risk in 77.8% of studies, some concerns in 20%, and high risk in 2.2%; (2) for deviations from intended interventions, 57.8% were at low risk, 26.7% some concerns, and 15.6% were high risk; (3) regarding missing outcome data, 88.9% were low risk, 8.9% some concerns, and 2.2% were high risk; (4) in the measurement of the outcome domain, 64.4% were rated as low risk, 22.2% as some concerns, and 13.3% as high risk; (5) for the selection of the reported result, 73.3% were at low risk, 24.4% some concerns, and 2.2% were high risk.

Deviations from the intended intervention were the domain with the highest level of bias across the studies. This high level of bias was primarily due to the lack of or inability to blind participants and/or intervention providers to the assigned study group. Only a few studies attempted to minimize this bias by blinding outcome assessors (50, 73, 90) and/or by blinding data analysis (80). Additionally, a second major source of bias in this domain stemmed from the high variability in implementing social component interventions across both groups. For the experimental group, this was observed when clowns, CLS, and/or caregivers employed flexible distraction strategies, and for the control group, when usual distraction methods were left to the discretion of healthcare personnel and caregivers. The interactive and adaptive nature of non-standardized social interventions increased the risk of deviations from the intended protocol, as the child's response, caregiver involvement, and intervention provider's style could differ significantly from case to case, impacting intervention consistency. Although some studies attempted to reduce variability in implementation by assigning the same provider, this approach increased the risk of bias in outcomes due to the lack of blinding, highlighting the methodological challenge of balancing standardization and control in studies with psychological interventions.

## Discussion

### Main findings and comparative effectiveness

This systematic review and meta-analysis compiled and analyzed the evidence available about interventions aimed at reducing pain, fear, and distress in children undergoing IMPs in PEDs, as assessed using self- or observer report or biomarkers. The interventions were grouped according to the type of stimulus, including somato-sensory, screen and immersive reality, social interaction, active command and unisensory distractions. The meta-analysis revealed the potential of psychological and sensory interventions to reduce pain, fear, and distress during the procedure, as well as induce some significant reductions in post-procedure biological markers. However, not all interventions demonstrated the same effectiveness across all outcomes.

Based on the size SMD, somato-sensory (Buzzy or Vibration, Cold Spray), tactile toys (Musical Bracelet Toy, Rotating Tactile Toy), and screen-based interventions (Informational Video, Cartoons) were the most effective interventions and exhibited concordance between child-reported and observer-reported outcomes for both pain and fear. Informational videos were the most effective interventions for both self-reported and observer-reported pain and fear outcomes, followed by rotating tactile toys in second place, and somato-sensory interventions, such as buzzy and cold spray, in third place. Active Command strategies were effective in pain and fear the more active the child's participation was. Regarding social interventions, finger puppet stood out as the most effective intervention for reducing observed distress, while interaction with a robot was only significant for self-reported pain. However, parental distraction, interaction with clowns, and CLS support did not show any significant results across all outcomes. VR-based interventions showed large discrepancies between child-reported and observer-reported outcomes for pain and fear. Interventions like VR video games and the Ceiling-Mounted VR Screen were not significant for self-reported outcomes but were significant for observer-reported pain and fear. In contrast, VR Glasses significantly reduced self-reported, but not observer-reported fear.

The large effect sizes observed in some analyses, and the discrepancies between observer- and self-reported outcomes, should be interpreted in light of methodological and contextual factors specific to PEDs settings. Observer ratings are particularly sensitive to visible behavioral changes, which can yield substantial standardized differences even with modest clinical improvements. In contrast, children's self-reports were often obtained after partial emotional regulation, resulting in smaller SMDs. Moreover, the high emotional reactivity typical of PEDs contexts may amplify observable changes in distress behavior, producing apparently larger intervention effects. These contextual influences indicate that large SMDs likely reflect not only the clinical efficacy of psychosensory interventions but also setting-specific perceptual and methodological factors.

### Mechanisms of psychosensory modulation

The findings of somato-sensory interventions in reducing pain and fear are align with previous research in primarily outpatient settings (92–95). Their effectiveness can be explained by the somatosensory activation of cutaneous nerve fibers that inhibit pain signals sent to the brain, Aβ fibers, which transmit sensations of touch, pressure, and vibration (Buzzy), and Aδ and C fibers, responsible for detecting stimuli related to pain and temperature (Cold Spray) (96, 97), facilitate an ascending modulation of pain (98). Concerning the difference in effect size between the two strategies on pain reduction (cold spray being more effective than Buzzy), recent evidence suggests that Cold Spray shows greater efficacy, as its inhibitory effect is more intense, rapid, and direct, whereas Buzzy modulates nociceptive signals without fully blocking them (99–102). Additionally, the combination of vibration with cold may cause both stimuli to compete for sensory attention, with the vibratory stimulus tending to dominate, diminishing the cold stimulus's effect and, thus, its efficacy in pain modulation (54, 103). On the other hand, the similar efficacy regarding fear levels can be understood from the conditioned inhibition perspective, where either intense acts as a safety signal, conditioning the child to associate the procedure with a less painful and threatening experience (104–106). However, our results also suggest informational videos and certain toys can be equally or more effective than somato-sensory, in the context of PEDs, which contrasts with outpatient settings where Buzzy consistently shows greater efficacy than other visual or auditory strategies (94). Specifically, the use of informational videos was the intervention with the greatest effect size in the meta-analysis. One key explanatory variable could be the influence of environmental stressors. A visit to an ED involves higher cognitive and emotional demands than outpatient care. These environmental stressors may act as amplifiers of fear experiences, increasing the relevance of a focus on modulating the cognitive and emotional pathways of pain (107). According to the cognitive and emotional pain modulation framework, pre-procedural education may act through cognitive restructuring of the experience, where information about what will happen (what, how, where, with whom) may help modulate the perception of threat and anticipatory emotional response (108–111). Taking into consideration that informational videos also show a child model successfully undergoing an IMP, their effectiveness can also be explained through vicarious learning processes, where a positive model can counteract fear acquired through negative experiences or previous negative models (112, 113).

Regarding the use of toys, three of them (Rotating Toy, Musical Bracelet, Kaleidoscope) were consistent with previous evidence in reducing pain and fear in outpatient settings (114–116). The fourth toy, MDT, did not reach statistical significance. This could be because, unlike the other three effective toys, which the child could touch or interact with during the procedure, the MDT could only be passively observed. From the perspective of pain neuroscience, tactile stimulus manipulation enhances the activation of descending pain modulation mechanisms through sensory and motor feedback (117–119), which may explain the differences in their effectiveness despite all being play objects.

Similarly, the effectiveness of active command or instruction strategies, where the child follows a guided action continuously during the procedure (e.g., squeezing a stress ball, blowing bubbles, palm stimulator and whistling), can be explained by both ascending and descending pain modulation. On the one hand, at the descending level the voluntary and repetitive nature of the action influences the activation of cognitive and emotional pain modulation areas. The types of actions themselves activate both sensory and autonomic systems. On the other hand, at the ascending level, similar to Buzzy and Cold Spray, the pressure of the stress ball and palm stimulator stimulates nerve fibers in the skin that can inhibit pain signals (120). Controlled breathing and bubble blowing activate sympathetic and parasympathetic systems, as a way of optimizing the modulation of psychophysiological pain responses (121–123).

The findings on the effectiveness of VR are consistent with mixed efficacy findings in predominantly outpatient settings (124–127). To gain a better insight into the heterogeneity observed in the effectiveness for VR interventions within meta-analysis, we consider four key variables that could explain the variation. Firstly, the age of the sample. During early to middle childhood, the proprioceptive and vestibular systems are still developing. Hence, VR interventions, which require high processing of both systems, increase the likelihood of discomfort with the device (128, 129), leading to variable efficacy of the experience on reducing pain, fear or distress. Particularly, studies involving infants and preschoolers showed more discomfort and a tendency to remove the device (78, 79). As half of the VR studies in the meta-analysis included early childhood participants, this could explain the lower efficacy outcomes across our meta-analysis. Secondly, environmental and illness-related stressors can impact the effectiveness of VR interventions. It has been documented that higher stress levels increase susceptibility to adverse VR effects (disorientation, dizziness, and nausea) (130). Both the stress of the ED environment and illness-related stress can limit participants' tolerance for VR, which varies widely among individuals, contributing to heterogeneous results across studies. Thirdly, the VR format and content varied widely amongst the included interventions. Among the 12 interventions using VR strategies, there were four presentation formats and 11 different content types. The level of cognitive and sensory load involved in each is key to its efficacy. The use of VR video games is considered the format with the highest cognitive demand due to the need for active interaction and real-time decision-making, which can affect children's ability to process and report their pain experience, leading to discrepancies in self-reported results (131–135). Lastly, the discrepancies between observed and self-perceived outcomes could have further added to the variation in effectiveness. Neuroscientific studies affirm that VR primarily influences visual and motor circuits, reducing behavioral pain expression, but to a lesser extent, it affects the anterior cingulate cortex and insula, which integrate the subjective dimension of pain (136). In high-cognitive-demand VR interventions, this could explain why observers perceive a behavioral change while participants may not experience a reduction in subjective pain. Conversely, low-cognitive-demand VR interventions may result in less behavioral pain expression but facilitate better subjective self-perception.

### Social and developmental factors

The effectiveness of social interventions in managing pain and distress in children during medical procedures appeared to be largely dependent on the child's age and the type of interaction. Finger puppet interaction in preschoolers was significant in reducing observed distress, likely due to the playful and dynamic nature of the interaction, which uses both visual and tactile stimulation, facilitating the modulation of cognitive and emotional pain pathways (95). In older children (6–12 years), the efficacy of interaction with robots on self-perceived pain could be associated with stimulating cognitive and emotional neural circuits, favoring downward pain regulation, which has also been evidenced in previous literature (137–140). On the other hand, parental distraction and CLS support, which did not show any significant impact across any of the outcomes, were applied to a broader sample (ages 2–16). Age differences may be important, considering that during early childhood, children are more dependent on parental co-regulation, while individual intervention strategies become more effective from school age onwards. Furthermore, evidence suggests that social pain modulation requires active and quality strategies (141, 142). In the ED context, not all caregivers may be effective in providing distraction after brief training, and parental stress levels may reduce the quality of their intervention (143–145). Importantly, none of the studies observed the parental or CLS's behaviors during the intervention, which would allow checking how well they were adhering to intervention training instructions rather than engaging in more habitual behaviors. Regarding the above, both verbal, nonverbal, and paraverbal elements of adult behavior have been suggested as influential variables to consider in the process of child co-regulation (146). Lastly, the exclusive effectiveness of clown therapy on self-reported fear levels could suggest that it primarily acts on cognitive or emotional pain pathways, rather than on nociceptive pain pathways (86, 147).

### Physiological and biological markers

The impact of the intervention on biomarkers of stress levels were mixed and largely dependent on the specific biomarker assessed and the timing of assessment, with the most impact observed post-intervention for heart rate and blood pressure. Informational videos were highly effective in reducing post-procedural heart rate and systolic and diastolic blood pressure (62), which is consistent with literature highlighting the sustained calming effects of cognitive preparation provided by informational videos (148, 149). This reduction in heart rate supports the notion that these videos promote parasympathetic recovery by helping children feel more prepared and less anxious about the procedure. This finding is consistent with previous studies were cognitive distraction through videos or educational content effectively lowered anxiety and, consequently, physiological stress markers such as blood pressure (150). Conversely, VR glasses significantly increased heart rate post-procedure (58). These mixed findings align with previous discussions on how VR can potentially induce sensory overload, which activates the sympathetic nervous system and raises heart rate (151, 152). Interestingly, the MDT also significantly increased heart rate post-procedure (86), which could be explained by the toy's engaging and dynamic nature, which provides distraction while potentially also increasing arousal in children, leading to sympathetic activation. Other interventions such as Buzzy or vibration, listening to music, and VR during the procedure showed no significant changes in heart rate or blood pressure, suggesting that their effects might be insufficient to counteract the autonomic responses to stress during the procedure itself (58, 73).

Lastly, limited evidence was available for SpO₂, respiratory rate, and plasma cortisol, showing inconclusive results for oxygen saturation and no significant alterations in plasma cortisol or respiratory rate. This highlights the need for more investigations on these biomarkers. The absence of significant effects on respiratory frequency (58) aligns with previous studies, where interventions such as distraction or sensory modulation failed to significantly affect respiratory frequency, possibly due to the short duration of the procedure or the mild intensity of the intervention (73, 150). Similarly, the lack of impact on cortisol levels could reflect the complexity of stress hormone modulation during medical interventions. On one hand, cortisol, a marker of hypothalamic–pituitary–adrenal (HPA) axis activation, may require more robust or prolonged interventions to produce significant changes, particularly in acute contexts such as medical procedures (144, 153). On the other hand, since the only study assessing cortisol levels used plasma samples, a method that may be less sensitive to subtle or transient stress responses compared with salivary or capillary cortisol, the absence of significant findings may also be due to methodological measurement factors.

### Clinical implications for PEDs

This systematic review and meta-analysis provide guiding results for the implementation of psychological and sensory interventions in PEDs. The main clinical implications, mechanisms of action, and recommended applications for each intervention type are summarized in Table 4. Protocols for effectively managing pain, fear, and distress in pediatric emergencies should consider the use of: (a) preparatory strategies (such as informational videos) to facilitate an initial modulation of the cognitive evaluation of the upcoming experience; (b) somato-sensory devices during venipuncture to activate the ascending pain modulation pathways; and (c) complementary distraction strategies, whether through the stimulation of cognitive and/or emotional processes that promote descending pain modulation. When selecting descending pain modulation interventions, it is recommended to consider the child's cognitive and sensory processing capacity, as well as the cognitive load involved in the intervention.

**Table 4:** Summary of intervention, mechanisms and key clinical implications for PEDs.

Type of intervention	Main mechanism	Clinical implications in PEDs
Somatosensory	Provide tactile and kinesthetic stimulation that modulates pain signals through sensory inhibition.	Strong and consistent reduction of pain and fear, particularly with Cold Spray and Buzzy/Vibration. Highly feasible, low-cost and quick to apply during IMPs
Screen-based	Direct audiovisual attention and modulate cognitive–emotional appraisal of threat.	Informational videos before an IMP are the most effective overall. It is useful when preparation time is available. Cartoons during procedures further support distraction.
Active command	Encourage rhythmic motor activity and breathing to support self-regulation and effective coping.	Simple, low-cost, and effective for reducing pain and fear when children actively participate during IMPs. Practical as a complementary strategy.
Toy interaction	Facilitate playful tactile or visual engagement that activates sensory feedback.	Effective for pain and fear. Useful when toys can be manipulated by the child. Best suited for brief IMPs, fostering positive engagement and distraction.
Immersive reality	Provide immersive multisensory distraction through visual and proprioceptive engagement.	Helpful for behavioral distress but effects on pain and fear are inconsistent. Limited by cost, setup time, and sensory tolerance. More suitable for older children and adolescents.
Social interaction	Engages emotional and social co-regulation to reduce perceived threat and enhance security cues.	Finger puppet play and robot interaction reduced distress, while parental and clown distraction yielded mixed results, likely influenced by caregiver stress and interaction quality.
Unisensory distractions	Use single-channel sensory input to focus attention (visual or auditory)	Music and card viewing showed moderate effects on fear and behavioral distress. Useful as low-cost complementary tools rather than standalone interventions.

PEDs, pediatric emergency departments; IMPs, invasive medical procedures.

In particular, the use of VR may overwhelm the vestibular and proprioceptive systems in infants and preschoolers. In these cases, simpler distraction strategies, such as screens, robots, or manipulable toys, are recommended. Further, at the preschooler age, sensory, emotional, and cognitive processes are dependent on adult behavior, highlighting the importance of involving caregivers with co-regulation strategies (154). Unfortunately, there is limited evidence regarding which specific co-regulation behaviors are effective in PED. For school-aged children, considering their still-developing systems, it is essential to make specific adjustments based on the level of interaction and type of VR content to ensure more effective pain and fear modulation (124, 127). In adolescents, there are less risk considerations related to their already developed sensory, vestibular, and proprioceptive systems (155). It is also important to allow adolescents to actively participate in choosing the intervention, as this promotes autonomy and a sense of control over their pain experience (156).

### Methodological limitations and future research

Despite the robustness of the findings, this meta-analysis is not without limitations. First, the high levels of heterogeneity observed indicate significant variability in study designs, intervention implementation, and outcome measures. This variability limits the generalizability of the findings and underscores the need for more standardized methodologies for both intervention design and outcome assessment in future research. Secondly, only articles published in English or Spanish were included, which could reduce the generalizability of the findings. Thirdly, the effectiveness of interventions on pain, fear, and distress outcomes was assessed exclusively during the invasive medical procedure, which may limit the interpretation of effects across different procedural phases. Interventions that did not yield significant results during the procedure might show efficacy in pre-procedural preparation or in facilitating post-procedural stress recovery. Fourth, most of the included studies do not include individual variables (previous pain experiences, central sensitivity, catastrophizing, attentional bias to pain, etc.) which could impact children's stress and the effectiveness of the interventions (28, 31–35, 157). Should all be considered cross the board to adopt a personalized approach that adjusts the intervention based on the patient's needs (158, 159).

Fifth, it is noteworthy that most interventions included in this review were implemented by healthcare professionals, with only one study (82) directly focusing on parental behaviors as the main component of the intervention. Conversely, only one study (69) explicitly excluded caregiver presence from the intervention group, considering that such presence could act as a non-pharmacological method to reduce children's anxiety and, therefore, represent a potential source of bias. This distinction underscores the importance of differentiating between active caregiver variables (behaviors) and passive variables (presence or absence). In this context, caregiver presence or absence emerges as a relevant yet scarcely explored factor, whose independent contribution to children's stress modulation warrants further investigation. Given that the inclusion criteria of this review prioritized active psychological and sensory interventions, future research should aim to disentangle the individual and combined effects of active caregiver behaviors and passive presence to better understand their respective roles in children's adaptive responses during IMPs.

Sixth, achieving blinding in psycho-sensorial interventions presents inherent challenges. The active involvement of children, caregivers, and healthcare professionals is integral to most interventions, making it practically impossible to fully blind the intervention's nature (160). Participants are consciously aware of both the activities performed and the intended objectives, which may introduce demand biases and skew outcomes positively. Social interventions, in particular, present added variability in delivery due to their interactive and adaptive nature. Given the difficulty of ensuring full blinding and standardizing application in psychological interventions, alternative methods are crucial for enhancing methodological rigor. Emphasizing blinding of outcome evaluators (50, 73, 90) and data analysts (80) may offer a feasible approach. Partial blinding strategies could also be used, where participants, although aware they are receiving an intervention, are not informed of the exact study objectives and hypotheses. Additionally, they may be kept unaware of whether they are in a specific “experimental” or “control” group when more than one intervention group or a control condition with minimally effective psychological or sensory strategies is included. For social interventions, consistent training of providers, structured response protocols for varying interaction scenarios, and fidelity assessments during intervention delivery can improve consistency and minimize potential deviations.

Seventh, although some studies reported physiological or biological, these were treated as secondary complementary outcomes to the main emotional or behavioral measures. This approach aligns with the review's focus on children's pain, fear, and distress as the core responses to procedural stress. While limited in scope, this inclusion allowed for an initial approximation of the physiological dimension of procedural stress and its potential modulation by psychosensory interventions. However, the available data were highly heterogeneous across studies, making it necessary to interpret these results with caution. Future research could benefit from integrating standardized physiological and biochemical measures to provide a more comprehensive psychophysiological understanding of stress regulation in pediatric IMPs.

Finally, given the high heterogeneity observed (*I*^2^ > 90%), results should be interpreted with caution. A random-effects model was applied *a priori* to address variability in intervention formats and study designs. Although subgroup analyses were pre-planned, only the classification by psychosensory subtype was feasible due to insufficient stratified data. In addition, no sensitivity or meta-regression analyses were conducted, which limits the assessment of result stability and potential moderators. This decision aimed to preserve statistical power given the small number of studies per category. Moreover, the geographical concentration of studies—primarily conducted in Turkey, Canada, and the United States—may have contributed modestly to between-study variability and limits the generalizability of findings to other healthcare contexts. Future meta-analyses with larger, more geographically diverse, and methodologically homogeneous datasets should include sensitivity and meta-regression analyses to explore sources of heterogeneity and confirm the robustness of these findings.

## Concluding remarks

In summary, this systematic review and meta-analysis suggest that a broad range of psychological and sensory interventions may reduce pain, fear, and distress during procedures in PED, as well as in some post-procedural biological markers. Pre-procedural preparation and procedural distraction appear to be key strategies for modulating these responses, addressing the sensory, cognitive, and emotional components of pediatric procedural pain. However, given the high heterogeneity among studies and the absence of meta-regression or sensitivity analyses, these findings should be interpreted with caution. Despite these limitations, the current evidence provides a preliminary useful foundation for developing clinical protocols aimed at optimizing the experience of children and adolescents in PED settings and minimizing long-term consequences, both psychological and somatosensory.

## Data Availability

The raw data supporting the conclusions of this article will be made available by the authors, without undue reservation.

## References

[1] Price J Kassam-Adams N Alderfer MA Christofferson J Kazak AE. Systematic review: a reevaluation and update of the integrative (trajectory) model of pediatric medical traumatic stress. J Pediatr Psychol. (2016) 41:86–97. 10.1093/jpepsy/jsv074 26319585

[2] Cozzi G Cognigni M Busatto R Grigoletto V Giangreco M Conte M Adolescents’ pain and distress during peripheral intravenous cannulation in a paediatric emergency setting. Eur J Pediatr. (2022) 181:125–31. 10.1007/s00431-021-04169-x 34218317 PMC8760195

[3] Hitchcock C Goodall B Wright IM Boyle A Johnston D Dunning D The early course and treatment of posttraumatic stress disorder in very young children: diagnostic prevalence and predictors in hospital-attending children and a randomized controlled proof-of-concept trial of trauma-focused cognitive therapy for 3- to 8-year-olds. J Child Psychol Psychiatry. (2022) 63:58–67. 10.1111/jcpp.13460 34128219

[4] Wingler D Liston D Joseph A Wang Y Feng H Martin L. Perioperative anxiety in pediatric surgery: induction room vs. operating room. Paediatr Anaesth. (2021) 31:465–73. 10.1111/pan.14098 33278852

[5] Marcev I Lannon-Boran C Hyland P McHugh Power J. The factors associated with paediatric medical post-traumatic stress: a systematic review. J Health Psychol. (2025) 30(11):2860–2880. 10.1177/13591053241272214 39344541 PMC12433540

[6] Ratnapalan S Cieslak P Mizzi T McEvoy J Mounstephen W. Physicians’ perceptions of background noise in a pediatric emergency department. Pediatr Emerg Care. (2011) 27:826–33. 10.1097/PEC.0b013e31822c1357 21878828

[7] Joseph MM Mahajan P Snow SK Ku BC Saidinejad M Optimizing pediatric patient safety in the emergency care setting. Pediatrics. (2022) 150:e2022058158. 10.1542/peds.2022-059673 36189487

[8] Haas R Brundisini F Barbara A Darvesh N Ritchie L MacDougall D Emergency department overcrowding: an environmental scan of contributing factors and a summary of systematic review evidence on interventions. Can J Health Technol. (2023) 3(11). 10.51731/cjht.2023.786 38320062

[9] Gripko M Joseph A MohammadiGorji S. Effects of the physical environment on children and families in hospital-based emergency departments: a systematic literature review. J Environ Psychol. (2023) 86:101970. 10.1016/j.jenvp.2023.101970 37366532 PMC10292152

[10] Gross TK Lane NE Timm NL Conners GP Hoffmann J Hsu B Crowding in the emergency department: challenges and recommendations for the care of children. Pediatrics. (2023) 151:e2022060603. 10.1542/peds.2022-060971 36808290

[11] Ortiz MI López-Zarco M Arreola-Bautista EJ. Procedural pain and anxiety in pediatric patients in a Mexican emergency department. J Adv Nurs. (2012) 68:2700–9. 10.1111/j.1365-2648.2012.05969.x 22381114

[12] Karlsson K Englund ACD Enskär K Parents’ perspectives on supporting children during needle-related medical procedures. Int J Qual Stud Health Well-being. (2014) 9:23759. 10.3402/qhw.v9.23759 25008196 PMC4090367

[13] Ringer T Moller D Mutsaers A. Distress in caregivers accompanying patients to an emergency department: a scoping review. J Emerg Med. (2017) 53:493–508. 10.1016/j.jemermed.2017.03.028 28499745

[14] Corrard F Copin C Wollner A Elbez A Derkx V Bechet S Sickness behavior in feverish children is independent of the severity of fever: an observational, multicenter study. PLoS One. (2017) 12:e0171670. 10.1371/journal.pone.0171670 28278190 PMC5344311

[15] Gates A Shave K Featherstone R Buckreus K Ali S Scott SD Procedural pain: systematic review of parent experiences and information needs. Clin Pediatr. (2018) 57:672–88. 10.1177/0009922817733694 28959897

[16] Martín SR Hung I Heyming TW Fortier MA Kain ZN. Predictors of parental anxiety in a paediatric emergency department. Emerg Med J. (2023) 40:715–20. 10.1136/emermed-2022-212917 37591685 PMC13221158

[17] Hellysaz A Neijd M Vesikari T Svensson L Hagbom M. Viral gastroenteritis: sickness symptoms and behavioral responses. mBio. (2023) 14:e0356722. 10.1128/mbio.03567-22 36976000 PMC10128049

[18] Birnie KA Noel M Chambers CT Uman LS Parker JA. Psychological interventions for needle-related procedural pain and distress in children and adolescents. Cochrane Database Syst Rev. (2020) 2020:CD005179. 10.1002/14651858.CD005179.pub4 PMC6517234 30284240

[19] Svendsen EJ Moen A Pedersen R Bjørk IT. Parent-healthcare provider interaction during peripheral vein cannulation with resistive preschool children. J Adv Nurs. (2016) 72:620–30. 10.1111/jan.12852 26577353

[20] Svendsen EJ Moen A Pedersen R Bjørk IT. Resistive expressions in preschool children during peripheral vein cannulation in hospitals: a qualitative explorative observational study. BMC Pediatr. (2015) 15:190. 10.1186/s12887-015-0508-3 26586285 PMC4653884

[21] Svendsen EJ Bjørk IT. Healthcare providers’ responses to children’s resistance to peripheral vein cannulation: a qualitative observational study. J Clin Nurs. (2021) 30:1325–34. 10.1111/jocn.15681 33529357

[22] Mitchell M Newall F Williams K. Behavioural emergencies in a paediatric hospital environment. J Paediatr Child Health. (2022) 58:1033–8. 10.1111/jpc.15896 35147266 PMC9305421

[23] Saidinejad M Duffy S Wallin D Hoffmann JA Joseph M Schieferle Uhlenbrock J The management of children and youth with pediatric mental and behavioral health emergencies. Ann Emerg Med. (2023) 82:e97–105. 10.1016/j.annemergmed.2023.06.003 37596031

[24] Shemesh E Keshavarz R Leichtling NK Weinberg E Mousavi A Sadow K Pediatric emergency department assessment of psychological trauma and posttraumatic stress. Psychiatr Serv. (2003) 54:1277–81. 10.1176/appi.ps.54.9.1277 12954946

[25] Pao M Bosk A. Anxiety in medically ill children/adolescents. Depress Anxiety. (2011) 28:40–9. 10.1002/da.20727 20721908 PMC2990785

[26] Simons LE. Fear of pain in children and adolescents with neuropathic pain and complex regional pain syndrome. Pain. (2016) 157:S90–7. 10.1097/j.pain.0000000000000377 26785161 PMC4721261

[27] Noel M Rosenbloom B Pavlova M Campbell F Isaac L Pagé MG Remembering the pain of surgery 1 year later: a longitudinal examination of anxiety in children’s pain memory development. Pain. (2019) 160:1729–39. 10.1097/j.pain.0000000000001582 31335643

[28] Hermann C Hohmeister J Demirakça S Zohsel K Flor H. Long-term alteration of pain sensitivity in school-aged children with early pain experiences. Pain. (2006) 125:278–85. 10.1016/j.pain.2006.08.026 17011707

[29] Cevik MÖ. Habituation, sensitization, and pavlovian conditioning. Front Integr Neurosci. (2014) 8:13. 10.3389/fnint.2014.00013 24574983 PMC3920081

[30] van den Hoogen NJ Patijn J Tibboel D Joosten BA Fitzgerald M Kwok CHT. Repeated touch and needle-prick stimulation in the neonatal period increases the baseline mechanical sensitivity and postinjury hypersensitivity of adult spinal sensory neurons. Pain. (2018) 159:1166–75. 10.1097/j.pain.0000000000001201 29528964 PMC5959002

[31] Woolf CJ. Central sensitization: implications for the diagnosis and treatment of pain. Pain. (2011) 152:S2–15. 10.1016/j.pain.2010.09.030 20961685 PMC3268359

[32] Baron R Hans G Dickenson AH. Peripheral input and its importance for central sensitization. Ann Neurol. (2013) 74:630–6. 10.1002/ana.24017 24018757

[33] Pas R Rheel E Van Oosterwijck S Leysen L Van De Vijver E Nijs J Endogenous pain modulation in children with functional abdominal pain disorders. Pain. (2019) 160:1883–90. 10.1097/j.pain.0000000000001566 31335656

[34] Fan W Sullivan SJ Sdrulla AD. Dorsal column and root stimulation at Aβ-fiber intensity activate superficial dorsal horn glutamatergic and GABAergic populations. Mol Pain. (2022) 18:17448069221079559. 10.1177/17448069221079559 35088625 PMC8891844

[35] Levy Gigi E Rachmani M Defrin R. The relationship between traumatic exposure and pain perception in children: the moderating role of posttraumatic symptoms. Pain. (2024) 165:2274–81. 10.1097/j.pain.0000000000003266 38728536

[36] Cunico D Rossi A Verdesca M Principi N Esposito S. Pain management in children admitted to the emergency room: a narrative review. Pharmaceuticals. (2023) 16:1178. 10.3390/ph16081178 37631093 PMC10459115

[37] Birnie KA Noel M Parker JA Chambers CT Uman LS Kisely SR Systematic review and meta-analysis of distraction and hypnosis for needle-related pain and distress in children and adolescents. J Pediatr Psychol. (2014) 39:783–808. 10.1093/jpepsy/jsu029 24891439 PMC4138805

[38] Case-Smith J Weaver LL Fristad MA. A systematic review of sensory processing interventions for children with autism spectrum disorders. Autism. (2015) 19:133–48. 10.1177/1362361313517762 24477447

[39] Camarata S Miller LJ Wallace MT. Evaluating sensory integration/sensory processing treatment: issues and analysis. Front Integr Neurosci. (2020) 14:556660. 10.3389/fnint.2020.556660 33324180 PMC7726187

[40] Ma D Su J Wang H Zhao Y Li H Li Y Sensory-based approaches in psychiatric care: a systematic mixed-methods review. J Adv Nurs. (2021) 77:3991–4004. 10.1111/jan.14884 33951221

[41] Lane AE. Practitioner review: effective management of functional difficulties associated with sensory symptoms in children and adolescents. J Child Psychol Psychiatry. (2020) 61:943–58. 10.1111/jcpp.13230 32166796

[42] Wan Yunus F Liu KP Bissett M Penkala S. Sensory-based intervention for children with behavioral problems: a systematic review. J Autism Dev Disord. (2015) 45:3565–79. 10.1007/s10803-015-2503-9 26092640

[43] Flor H. Psychological pain interventions and neurophysiology: implications for a mechanism-based approach. Am Psychol. (2014) 69:188–96. 10.1037/a0035254 24547804

[44] Mahmood A Hunt N Masiewicz S Cranford JA Noel S Brent C Treating prehospital pain in children: a retrospective chart review comparing the safety and efficacy of prehospital pediatric ketamine and opioid analgesia. J Pain Palliat Care Pharmacother. (2023) 37:133–42. 10.1080/15360288.2023.2169433 36716228

[45] Page MJ McKenzie JE Bossuyt PM Boutron I Hoffmann TC Mulrow CD The PRISMA 2020 statement: an updated guideline for reporting systematic reviews. Br Med J. (2021) 372:n71. 10.1136/bmj.n71 33782057 PMC8005924

[46] Higgins JPT Altman DG Sterne JAC. Chapter 8: Assessing risk of bias in included studies. In: Higgins JPT Green S, editors. Cochrane Handbook for Systematic Reviews of Interventions. Version 5.1.0. the Cochrane Collaboration. London: The Cochrane Collaboration (2011). p. d5928. Available online at: https://www.cochrane.org/authors/handbooks-and-manuals/handbook/current/chapter-08

[47] Baxter AL Cohen LL McElvery HL Lawson ML von Baeyer CL. An integration of vibration and cold relieves venipuncture pain in a pediatric emergency department. Pediatr Emerg Care. (2011) 27:1151–6. 10.1097/PEC.0b013e318237ace4 22134226

[48] Çelik EG Sönmez Düzkaya D. The impact of cold spray and ice application during intravenous access on pain and fear in children aged 7–15 years in the pediatric emergency unit: a randomized controlled trial. J Emerg Nurs. (2024) 50:264–72. 10.1016/j.jen.2023.11.012 38142386

[49] Düzkaya DS Karakul A Akoy İ Sönmez Düzkaya D Andi S. Effects of ShotBlocker® and the Helfer skin tap technique on pain and fear experienced during intramuscular injection among children aged 6–12 years in pediatric emergency units: a randomized controlled trial. Int Emerg Nurs. (2024) 76:101502. 10.1016/j.ienj.2024.101502 39126884

[50] Farion KJ Splinter KL Newhook K Gaboury I. The effect of vapocoolant spray on pain due to intravenous cannulation in children: a randomized controlled trial. Can Med Assoc J. (2008) 179:31–6. 10.1503/cmaj.070874 18591524 PMC3267474

[51] Girgin BA Aktaş E Kılınç D Aykanat Girgin B Gözen D. Let’s prefer the pain reducing intervention, Buzzy or ShotBlocker: a randomized controlled trial. J Pediatr Nurs. (2020) 51:75–83. 10.5222/buchd.2020.1300731926405

[52] Haidar NA Al Amri MH Sendad NG Toaimah FHS. Efficacy of Buzzy device versus EMLA cream for reducing pain during needle-related procedures in children: a randomized controlled trial. Pediatr Emerg Care. (2024) 40:180–6. 10.1097/PEC.0000000000002965 37163686

[53] Halal Mehdi Alfatavi H Sadeghi T Baqer Hassan Mohammed Al-Dakheel M Effects of whistling compared with Buzzy device during blood sampling on pain and fear in children’s emergency department. Compr Child Adolesc Nurs. (2022) 45:414–24. 10.1080/24694193.2022.2091683 36440867

[54] Semerci R Akarsu Ö Kılıç D. The effect of Buzzy and cold spray on pain, anxiety, and fear of children during venipuncture in pediatric emergency department in Turkey: a randomized controlled study. J Pediatr Nurs. (2023) 68:e1–7. 10.1016/j.pedn.2022.08.019 36089558

[55] Sivri Bilgen B Balcı S. The effect on pain of Buzzy® and ShotBlocker® during the administration of intramuscular injections to children: a randomized controlled trial. J Korean Acad Nurs. (2019) 49:486–94. 10.4040/jkan.2019.49.4.486 31477677

[56] Sivri BB Balcı S Dolgun G. The effect of 3 methods (Buzzy, ShotBlocker, and DistrACTION cards) used while taking blood samples from children with pain and anxiety: a randomized controlled trial. Pediatr Emerg Care. (2023) 39:600–7. 10.1097/PEC.0000000000002866 36730932

[57] Uzsen H Tural Buyuk E Odabasoglu E Koyun M. The effects of vibration and pressure interventions on children’s pain, fear, and anxiety: a randomized controlled trial. J Pediatr Nurs. (2024) 75:196–204. 10.1016/j.pedn.2023.12.022 38171061

[58] Yıldırım BG Gerçeker GÖ. The effect of virtual reality and Buzzy on first insertion success, procedure-related fear, anxiety, and pain in children during intravenous insertion in the pediatric emergency unit: a randomized controlled trial. J Emerg Nurs. (2023) 49:62–74. 10.1016/j.jen.2022.09.018 36376127

[59] Yilmaz G Alemdar DK. Using Buzzy, ShotBlocker, and bubble blowing in a pediatric emergency department to reduce the pain and fear caused by intramuscular injection: a randomized controlled trial. J Emerg Nurs. (2019) 45:502–11. 10.1016/j.jen.2019.04.003 31257044

[60] Zengin M Yayan EH. A comparison of two different tactile stimulus methods on reducing pain of children during intramuscular injection: a randomized controlled study. J Emerg Nurs. (2022) 48:167–80. 10.1016/j.jen.2021.10.006 34952709

[61] Daihimfar F Babamohamadi H Ghorbani R. A comparison of the effects of acupressure and music on venipuncture pain intensity in children: a randomized controlled clinical trial. Pain Res Manag. (2024) 2024:2504732. 10.1155/2024/2504732 38274399 PMC10810694

[62] Düzkaya DS Bozkurt G Ulupınar S Uysal G Uçar S Uysalol M. The effect of a cartoon and an information video about intravenous insertion on pain and fear in children aged 6 to 12 years in the pediatric emergency unit: a randomized controlled trial. J Emerg Nurs. (2021) 47:76–87. 10.1016/j.jen.2020.04.011 32690314

[63] Lee HN Park JW Hwang S Jung JY Kim DK Kwak YH. Effect of a virtual reality environment using a domed ceiling screen on procedural pain during intravenous placement in young children: a randomized clinical trial. JAMA Pediatr. (2023) 177:25–31. 10.1001/jamapediatrics.2022.4426 36409508 PMC9679961

[64] Miller K Tan X Hobson AD Khan A Ziviani J O'Brien E A prospective randomized controlled trial of nonpharmacological pain management during intravenous cannulation in a pediatric emergency department. Pediatr Emerg Care. (2016) 32:444–51. 10.1097/PEC.0000000000000778 27380603

[65] Akarsu Ö Semerci R Kılınç D. The effect of two different distraction methods on pain, fear, and anxiety levels during venous blood draw in children in a pediatric emergency unit: a randomized controlled study. J Nurs Care Qual. (2023) 38:E51–8. 10.1097/NCQ.0000000000000709 36943230

[66] Ali S Ma K Dow N Vandermeer B Scott S Beran T A randomized trial of iPad distraction to reduce children’s pain and distress during intravenous cannulation in the paediatric emergency department. Paediatr Child Health. (2021) 26:287–93. 10.1093/pch/pxaa089 34630780 PMC8496185

[67] Downey LV Zun LS. The impact of watching cartoons for distraction during painful procedures in the emergency department. Pediatr Emerg Care. (2012) 28:1033–5. 10.1097/PEC.0b013e31826cac1a 23023471

[68] Dumoulin S Bouchard S Ellis J Lavoie KL Vézina M-P Charbonneau P A randomized controlled trial on the use of virtual reality for needle-related procedures in children and adolescents in the emergency department. Games Health J. (2019) 8:285–93. 10.1089/g4h.2018.0111 31135178

[69] Míguez-Navarro C. Video distraction system to reduce anxiety and pain in children subjected to venipuncture in pediatric emergencies. Pediatr Emerg Care Med. (2016) 1(1):1–4.

[70] van der Heijden MJE Mevius H van der Heijde N van Rosmalen J van As S van Dijk M. Children listening to music or watching cartoons during ER procedures: a RCT. J Pediatr Psychol. (2019) 44:1151–62. 10.1093/jpepsy/jsz066 31621845

[71] Arıkan A Esenay FI. Active and passive distraction interventions in a pediatric emergency department to reduce the pain and anxiety during venous blood sampling: a randomized clinical trial. J Emerg Nurs. (2020) 46:779–90. 10.1016/j.jen.2020.05.004 32711948

[72] Goktas N Avci D. The effect of visual and/or auditory distraction techniques on children’s pain, anxiety and medical fear in invasive procedures: a randomized controlled trial. J Pediatr Nurs. (2023) 73:e27–35. 10.1016/j.pedn.2023.07.005 37455147

[73] Hartling L Newton AS Liang Y Jou H Hewson K Klassen TP Music to reduce pain and distress in the pediatric emergency department: a randomized clinical trial: a randomized clinical trial. JAMA Pediatr. (2013) 167:826–35. 10.1001/jamapediatrics.2013.200 23857075

[74] Can M Özalp Gerçeker G. The effect of the veinlite PEDI2 and passive virtual reality distraction on peripheral catheter insertion-related emotional behavior, pain, fear, and anxiety of children: a randomized controlled trial. J Pediatr Nurs. (2024) 78:e227–35. 10.1016/j.pedn.2024.07.010 39060169

[75] Chan E Hovenden M Ramage E Ling N Pham JH Rahim A Virtual reality for pediatric needle procedural pain: two randomized clinical trials. J Pediatr. (2019) 209:160–7.e4. 10.1016/j.jpeds.2019.02.034 31047650

[76] Chen YJ Cheng SF Lee PC Lai C Hou I. Distraction using virtual reality for children during intravenous injections in an emergency department: a randomised trial. J Clin Nurs. (2020) 29:503–10. 10.1111/jocn.15088 31715039

[77] Goldman RD Behboudi A. Virtual reality for intravenous placement in the emergency department: a randomized controlled trial. Eur J Pediatr. (2021) 180:725–31. 10.1007/s00431-020-03771-9 32779029

[78] Osmanlliu E Trottier ED Bailey B Lagacé M Certain M Khadra C Distraction in the emergency department using virtual reality for intravenous procedures in children to improve comfort (DEVINCI): a pilot pragmatic randomized controlled trial. Can J Emerg Med. (2021) 23:94–102. 10.1007/s43678-020-00006-6 33683617

[79] Schlechter AK Whitaker W Iyer S Gabriele G Wilkinson M. Virtual reality distraction during pediatric intravenous line placement in the emergency department: a prospective randomized comparison study. Am J Emerg Med. (2021) 44:296–9. 10.1016/j.ajem.2020.04.009 32307295

[80] Oluc N Tas Arslan F. The effect of two different methods on reducing the pain and fear during phlebotomy in children: a randomized controlled trial. Int Emerg Nurs. (2024) 72:101386. 10.1016/j.ienj.2023.101386 37984025

[81] Ali S Manaloor R Ma K Sivakumar M Beran T Scott SD A randomized trial of robot-based distraction to reduce children’s distress and pain during intravenous insertion in the emergency department. Can J Emerg Med. (2021) 23:85–93. 10.1007/s43678-020-00023-5 33683608

[82] Cavender K Goff MD Hollon EC Guzzetta CE. Parents’ positioning and distracting children during venipuncture: effects on children’s pain, fear, and distress. J Holist Nurs. (2004) 22:32–56. 10.1177/0898010104263306 15035240

[83] Felluga M Rabach I Minute M Montico M Giorgi R Lonciari I A quasi randomized-controlled trial to evaluate the effectiveness of clown therapy on children’s anxiety and pain levels in emergency department. Eur J Pediatr. (2016) 175:645–50. 10.1007/s00431-015-2688-0 26755209

[84] Rimon A Shalom S Wolyniez I Gruber A Schachter-Davidov A Glatstein M. Medical clowns and cortisol levels in children undergoing venipuncture in the emergency department: a pilot study. Isr Med Assoc J. (2016) 18(11):680–3. 28466619

[85] Wolyniez I Rimon A Scolnik D Gruber A Tavor O Haviv E The effect of a medical clown on pain during intravenous access in the pediatric emergency department: a randomized prospective pilot study. Clin Pediatr. (2013) 52:1168–72. 10.1177/0009922813502257 24028842

[86] Karaca TN Cevik Guner U. The effect of music-moving toys to reduce fear and anxiety in preschool children undergoing intravenous insertion in a pediatric emergency department: a randomized clinical trial. J Emerg Nurs. (2022) 48:32–44. 10.1016/j.jen.2021.10.004 34865858

[87] Lilik Lestari MP Wanda D Hayati H. The effectiveness of distraction (cartoon-patterned clothes and bubble-blowing) on pain and anxiety in preschool children during venipuncture in the emergency department. Compr Child Adolesc Nurs. (2017) 40:22–8. 10.1080/24694193.2017.1386967 29166202

[88] Şen T Çetinkaya B. The effect of virtual reality glasses used during intravenous catheter application on the child’s emotional responses. J Pediatr Nurs. (2024) 77:e251–6. 10.1016/j.pedn.2024.04.036 38692952

[89] Ceylan M Erkut Z. The effect of finger puppet on pain and emotional manifestation for venous blood collection in the pediatric emergency department: a randomized controlled trial. Int Emerg Nurs. (2023) 70:101348. 10.1016/j.ienj.2023.101348 37708789

[90] Stevenson MD Bivins CM O’Brien K O'Brien K Gonzalez del Rey JA. Child life intervention during angiocatheter insertion in the pediatric emergency department. Pediatr Emerg Care. (2005) 21:712–8. 10.1097/01.pec.0000186423.84764.5a 16280943

[91] Bourdier S Khelif N Velasquez M Usclade A Rochette E Pereira B Cold vibration (Buzzy) versus anesthetic patch (EMLA) for pain prevention during cannulation in children: a randomized trial. Pediatr Emerg Care. (2021) 37:86–91. 10.1097/PEC.0000000000001867 31181022

[92] Bergomi P Scudeller L Pintaldi S Dal Molin A. Efficacy of non-pharmacological methods of pain management in children undergoing venipuncture in a pediatric outpatient clinic: a randomized controlled trial of audiovisual distraction and external cold and vibration. J Pediatr Nurs. (2018) 42:e66–72. 10.1016/j.pedn.2018.04.011 29728296

[93] Ballard A Khadra C Adler S Trottier ED Le May S. Efficacy of the Buzzy device for pain management during needle-related procedures: a systematic review and meta-analysis. Clin J Pain. (2019) 35:532–43. 10.1097/AJP.0000000000000690 30829735

[94] Jin F Wang X Qi M Zhang W Zhang Y. Effectiveness and safety of Buzzy device in needle-related procedures for children under twelve years of age: a systematic review and meta-analysis. Medicine. (2024) 103:e37522. 10.1097/MD.0000000000037522 38608108 PMC11018245

[95] Yaz ŞB Başdemir S Geçtan E. The effect of vibrating cold application and puppet use on pain and fear during phlebotomy in children: a randomized controlled study. J Pediatr Nurs. (2024) 74:77–84. 10.1016/j.pedn.2023.11.018 38029689

[96] Schmelz M. Neuronal sensitivity of the skiN.Eur J Dermatol. (2011) 2:43–7. 10.1684/ejd.2011.1265 21628129

[97] Sonza A Sanada LS Oliveira LR Bernardo-Filho M Sá-Caputo DC Zaro MA Whole-body vibration mediates mechanical hypersensitivity through a*β*-fiber and C-fiber thermal sensation in a chronic pain model. Exp Biol Med. (2021) 246:1210–8. 10.1177/1535370221991147 PMC8142106 33593110

[98] Tobaldini G Sardi NF Guilhen VA Fischer L. Pain inhibits pain: an ascending-descending pain modulation pathway linking mesolimbic and classical descending mechanisms. Mol Neurobiol. (2019) 56:1000–13. 10.1007/s12035-018-1116-7 29858776

[99] Defrin R Sheraizin A Malichi L Shachen O. Spatial summation and spatial discrimination of cold pain: effect of spatial configuration and skin type. Pain. (2011) 152:2739–45. 10.1016/j.pain.2011.08.017 21959364

[100] Selvi F Bedel C Akçimen M. Evaluation of vapocoolant spray effect on pain reduction during digital nerve block: a randomized clinical trial. Am J Emerg Med. (2021) 50:260–3. 10.1016/j.ajem.2021.08.001 34418716

[101] Shimo K Ogawa S Niwa Y Tokiwa Y Dokita A Kato S Inhibition of current perception thresholds in A-delta and C fibers through somatosensory stimulation of the body surface. Sci Rep. (2022) 12:13705. 10.1038/s41598-022-18016-y 35962024 PMC9374682

[102] Pakalniskis J Soares S Rajan S Vyshnevska A Schmelz M Solinski HJ Human pain ratings to electrical sinusoids increase with cooling through a cold-induced increase in C-fibre excitability. Pain. (2023) 164:1524–36. 10.1097/j.pain.0000000000002849 36972485

[103] Hollins M McDermott K Harper D. How does vibration reduce pain? Perception. (2014) 43:70–84. 10.1068/p7637 24689133

[104] Wood KH Ver Hoef LW Knight DC. Neural mechanisms underlying the conditioned diminution of the unconditioned fear response. Neuroimage. (2012) 60:787–99. 10.1016/j.neuroimage.2011.12.048 22227141

[105] Odriozola P Gee DG. Learning about safety: conditioned inhibition as a novel approach to fear reduction targeting the developing brain. Am J Psychiatry. (2021) 178:136–55. 10.1176/appi.ajp.2020.20020232 33167673 PMC7951569

[106] Laing PAF Vervliet B Dunsmoor JE Harrison BJ. Pavlovian safety learning: an integrative theoretical review. Psychon Bull Rev. (2025) 32:176–202. 10.3758/s13423-024-02559-4 39167292

[107] Bushnell MC Čeko M Low LA. Cognitive and emotional control of pain and its disruption in chronic pain. Nat Rev Neurosci. (2013) 14:502–11. 10.1038/nrn3516 23719569 PMC4465351

[108] Hodgins MJ Lander J. Children’s coping with venipuncture. J Pain Symptom Manage. (1997) 13:274–85. 10.1016/s0885-3924(96)00328-4 9185433

[109] Wang Y Jackson T Cai L. Causal effects of threat and challenge appraisals on coping and pain perceptioN.Eur J Pain. (2016) 20:1111–20. 10.1002/ejp.835 26773726

[110] Jamieson JP Hangen EJ Lee HY Yeager DS. Capitalizing on appraisal processes to improve affective responses to social stress. Emot Rev. (2018) 10:30–9. 10.1177/1754073917693085 31178923 PMC6550483

[111] Wiech K Ploner M Tracey I. Neurocognitive aspects of pain perception. Trends Cogn Sci. (2008) 12:306–13. 10.1016/j.tics.2008.05.005 18606561

[112] Askew C Field AP. Vicarious learning and the development of fears in childhood. Behav Res Ther. (2007) 45:2616–27. 10.1016/j.brat.2007.06.008 17651688

[113] Krause L Askew C. Preventing and reducing fear using positive modelling: a systematic review of experimental research with children. Behav Res Ther. (2022) 148:103992. 10.1016/j.brat.2021.103992 34837839

[114] Turgut MA Türkmen AS. The effect of lighted toy on reducing pain and fear during blood collection in children between 3 and 6 years: a randomized control trial. J Pediatr Nurs. (2023) 70:111–6. 10.1016/j.pedn.2023.02.009 36905910

[115] Koç S Küçük Alemdar D. Effect of a musical toy used during peripheral venous access on children’s pain, fear and parental satisfaction: randomized controlled trial. J Pediatr Nurs. (2024) 77:e573–82. 10.1016/j.pedn.2024.05.024 38821765

[116] Taspinar F Turkmen AS. The impact of kaleidoscope on children’s pain and fear during sutures. Int Emerg Nurs. (2024) 77:101521. 10.1016/j.ienj.2024.101521 39342773

[117] Mancini F Beaumont A-L Hu L Haggard P Iannetti GDD. Touch inhibits subcortical and cortical nociceptive responses. Pain. (2015) 156:1936–44. 10.1097/j.pain.0000000000000253 26058037 PMC4579551

[118] Zhao K Tang Z Wang H Guo Y Peng W Hu L. Analgesia induced by self-initiated electrotactile sensation is mediated by top-down modulations. Psychophysiology. (2017) 54:848–56. 10.1111/psyp.12839 28169425

[119] Bannister K Dickenson AH. The plasticity of descending controls in pain: translational probing. J Physiol. (2017) 595:4159–66. 10.1113/JP274165 28387936 PMC5491855

[120] Blanc C Buisson J-C Kruck J Kostrubiec V. Haptic coordination: squeezing a vibrating stress ball decreases anxiety and arousal. Hum Mov Sci. (2024) 96:103220. 10.1016/j.humov.2024.103220 38776797

[121] Fincham GW Strauss C Montero-Marin J Cavanagh K. Effect of breathwork on stress and mental health: a meta-analysis of randomised-controlled trials. Sci Rep. (2023) 13:432. 10.1038/s41598-022-27247-y 36624160 PMC9828383

[122] Chin MS Kales SN. Is there an optimal autonomic state for enhanced flow and executive task performance? Front Psychol. (2019) 10:1716. 10.3389/fpsyg.2019.01716 31474898 PMC6702786

[123] Buchanan TL Janelle CM. Emotions and ensuing motor performance are altered by regulating breathing frequency: implications for emotion regulation and sport performance. Front Psychol. (2022) 13:963711. 10.3389/fpsyg.2022.963711 36275219 PMC9582930

[124] Merino-Lobato C Rodríguez-Gallego I Pabón-Carrasco M Romero-Castillo R Jiménez-Picón N. Virtual reality vs. Buzzy®. efficacy in pain and anxiety management during pediatric venipuncture. Systematic review and meta-analysis. J Pediatr Nurs. (2023) 73:22–33. 10.1016/j.pedn.2023.08.014 37603924

[125] Guillari A Giordano V Catone M Gallucci M Rea T. Non-pharmacological interventions to reduce procedural needle pain in children (6-12 years): a systematic review. J Pediatr Nurs. (2024) 78:e102–16. 10.1016/j.pedn.2024.06.025 39013701

[126] Hu Z Yao J He L Guo Y. The impact of virtual reality exposure on anxiety and pain levels in pediatric patients: a systematic review and meta-analysis. J Pediatr Nurs. (2024) 78:e364–74. 10.1016/j.pedn.2024.07.027 39085008

[127] Savaş EH Demir AS Semerci R Karadağ A. Effect of virtual reality on pain during burn dressing in children: a systematic review and meta-analysis of randomized controlled trials. J Pediatr Nurs. (2023) 73:e364–71. 10.1016/j.pedn.2023.10.002 37806856

[128] Tychsen L Foeller P. Effects of immersive virtual reality headset viewing on young children: visuomotor function, postural stability, and motion sickness. Am J Ophthalmol. (2020) 209:151–9. 10.1016/j.ajo.2019.07.020 31377280

[129] Valori I McKenna-Plumley PE Bayramova R Zandonella Callegher C Altoè G Farroni T. Proprioceptive accuracy in immersive virtual reality: a developmental perspective. PLoS One. (2020) 15:e0222253. 10.1371/journal.pone.0222253 31999710 PMC6992210

[130] Nalivaiko E Davis SL Blackmore KL Vakulin A Nesbitt KV. Cybersickness provoked by head-mounted display affects cutaneous vascular tone, heart rate and reaction time. Physiol Behav. (2015) 151:583–90. 10.1016/j.physbeh.2015.08.043 26340855

[131] Hoffman HG. Interacting with virtual objects via embodied avatar hands reduces pain intensity and diverts attention. Sci Rep. (2021) 11:10672. 10.1038/s41598-021-89526-4 34021173 PMC8140079

[132] Li J Yang H Xiao Y Liu X. The analgesic effects and neural oscillatory mechanisms of virtual reality scenes based on distraction and mindfulness strategies in human volunteers. Br J Anaesth. (2023) 131:1082–92. 10.1016/j.bja.2023.09.001 37798154

[133] Lobel A Granic I Stone LL Engels RCME. Associations between children’s video game playing and psychosocial health: information from both parent and child reports. Cyberpsychol Behav Soc Netw. (2014) 17:639–43. 10.1089/cyber.2014.0128 25272237

[134] Verhoef REJ van Dijk A Verhulp EE de Castro BO. Interactive virtual reality assessment of aggressive social information processing in boys with behaviour problems: a pilot study. Clin Psychol Psychother. (2021) 28:489–99. 10.1002/cpp.2620 34048619 PMC8361679

[135] da Silva Soares R Jr Ramirez-Chavez KL Tufanoglu A Barreto C Sato JR Ayaz H. Cognitive effort during visuospatial problem solving in physical real world, on computer screen, and in virtual reality. Sensors. (2024) 24:977. 10.3390/s24030977 38339693 PMC10857420

[136] Mehesz E Karoui H Strutton PH Hughes SW. Exposure to an immersive virtual reality environment can modulate perceptual correlates of endogenous analgesia and central sensitization in healthy volunteers. J Pain. (2021) 22:707–14. 10.1016/j.jpain.2020.12.007 33465506

[137] Rossi S Santini SJ Di Genova D Maggi G Verrotti A Farello G Using the social robot NAO for emotional support to children at a pediatric emergency department: randomized clinical trial. J Med Internet Res. (2022) 24:e29656. 10.2196/29656 34854814 PMC8796042

[138] Beran TN Ramirez-Serrano A Vanderkooi OG Kuhn S. Reducing children’s pain and distress towards flu vaccinations: a novel and effective application of humanoid robotics. Vaccine. (2013) 31:2772–7. 10.1016/j.vaccine.2013.03.056 23623861

[139] Okita SY. Self-other’s perspective taking: the use of therapeutic robot companions as social agents for reducing pain and anxiety in pediatric patients. Cyberpsychol Behav Soc Netw. (2013) 16:436–41. 10.1089/cyber.2012.0513 23505968

[140] Lee-Krueger RCW Pearson JR Spencer A Noel M Bell-Graham L Beran TN. Children’s pain during IV induction: a randomized-controlled trial with the MEDi® robot. J Pediatr Psychol. (2021) 46:991–1000. 10.1093/jpepsy/jsab028 33764470

[141] Che X Cash R Chung S Fitzgerald PB Fitzgibbon BM. Investigating the influence of social support on experimental pain and related physiological arousal: a systematic review and meta-analysis. Neurosci Biobehav Rev. (2018) 92:437–52. 10.1016/j.neubiorev.2018.07.005 30006033

[142] Roberts MH Klatzkin RR Mechlin B. Social support attenuates physiological stress responses and experimental pain sensitivity to cold pressor pain. Ann Behav Med. (2015) 49:557–69. 10.1007/s12160-015-9686-3 25623896

[143] McCarthy AM Kleiber C Hanrahan K Zimmerman MB Westhus N Allen S. Impact of parent-provided distraction on child responses to an IV insertion. Child Health Care. (2010) 39:125–41. 10.1080/02739611003679915 21643530 PMC3106296

[144] McCarthy AM Kleiber C Hanrahan K Zimmerman MB Ersig A Westhus N Matching doses of distraction with child risk for distress during a medical procedure: a randomized clinical trial: a randomized clinical trial. Nurs Res. (2014) 63:397–407. 10.1097/NNR.0000000000000056 25350539 PMC4282990

[145] Newell A Keane J McGuire BE Heary C McDarby V Dudley B Interactive versus passive distraction and parent psychoeducation as pain management techniques during pediatric venepuncture: a randomized controlled trial. Clin J Pain. (2018) 34:1008–16. 10.1097/ajp.0000000000000628 29750665

[146] McMurtry MC Chambers CT McGrath PJ Asp E. When “don’t worry” communicates fear: children’s perceptions of parental reassurance and distraction during a painful medical procedure. Pain. (2010) 150:52–8. 10.1016/j.pain.2010.02.021 20227831

[147] Meiri N Ankri A Hamad-Saied M Konopnicki M Pillar G. The effect of medical clowning on reducing pain, crying, and anxiety in children aged 2-10 years old undergoing venous blood drawing–a randomized controlled study. Eur J Pediatr. (2016) 175:373–9. 10.1007/s00431-015-2652-z 26475347

[148] Doering S Katzlberger F Rumpold G Roessler S Hofstoetter B Schatz DS Videotape preparation of patients before hip replacement surgery reduces stress. Psychosom Med. (2000) 62:365–73. 10.1097/00006842-200005000-00010 10845350

[149] Liu Y Chen J Pan Y Cai Y Ge C Chu H The effects of video based nursing education on perioperative anxiety and depression in patients with gastric cancer. Psychol Health Med. (2021) 26:867–76. 10.1080/13548506.2020.1825756 33044837

[150] Sogabe M Okahisa T Fukuya A Kagemoto K Okada Y Adachi Y Effects of audio and visual distraction on patients’ vital signs and tolerance during esophagogastroduodenoscopy: a randomized controlled trial. BMC Gastroenterol. (2020) 20:122. 10.1186/s12876-020-01274-3 32316918 PMC7175521

[151] Gao Y Wang N Liu N.E ffectiveness of virtual reality in reducing preoperative anxiety in adults: a systematic review and meta-analysis. J Adv Nurs. (2023) 79:3678–90. 10.1111/jan.15743 37350039

[152] Qin Z Zhou C Zhu Y Wang Y Cao H Huang Z. Virtual reality for hypertension in tooth extraction: a randomized trial. J Dent Res. (2022) 101:400–6. 10.1177/00220345211049393 34825613

[153] Rogerson O Wilding S Prudenzi A O’Connor DB. Effectiveness of stress management interventions to change cortisol levels: a systematic review and meta-analysis. Psychoneuroendocrinology. (2024) 159:106415. 10.1016/j.psyneuen.2023.106415 37879237

[154] Lunoe MM Bolin AE Drendel AL. An evaluation of high preprocedural anxiety and venipuncture pain experienced by young children. Pediatr Emerg Care. (2021) 37:e621–4. 10.1097/PEC.0000000000002424 34591812

[155] Blankenburg M Boekens H Hechler T Maier C Krumova E Scherens A Reference values for quantitative sensory testing in children and adolescents: developmental and gender differences of somatosensory perception. Pain. (2010) 149:76–88. 10.1016/j.pain.2010.01.011 20138430

[156] Riggenbach A Amouroux R Van Petegem S Tourniaire B Tonelli A Wiener S Autonomy and competence satisfaction as resources for facing chronic pain disability in adolescence: a self-determination perspective: original article. Psychol Health Med. (2021) 26:322–32. 10.1080/13548506.2020.1813900 32865427

[157] Vervoort T Trost Z Van Ryckeghem DML. Children’s selective attention to pain and avoidance behaviour: the role of child and parental catastrophizing about pain. Pain. (2013) 154(10):1979–88. 10.1016/j.pain.2013.05.052 23792243

[158] Richardson PA Birnie KA Harrison LE Rajagopalan A Bhandari RP. Profiling modifiable psychosocial factors among children with chronic pain: a person-centered methodology. J Pain. (2020) 21(3–4):467–76. 10.1016/j.jpain.2019.08.015 31521795

[159] McCracken LM. Personalized pain management: is it time for process-based therapy for particular people with chronic pain? Eur J Pain. (2023) 27(9):1044–55. 10.1002/ejp.2091 36755478

[160] Boutron I Moher D Altman DG Schulz KF Ravaud P, CONSORT Group. Extending the CONSORT statement to randomized trials of nonpharmacologic treatment: explanation and elaboration. Ann Intern Med. (2008) 148(4):295–309. 10.7326/0003-4819-148-4-200802190-00008 18283207

