# Supplementary figures and images for: Efficacy of psychosensory interventions for the management of pediatric pain, fear, and distress during emergency care: a systematic review and meta-analysis of randomized clinical trials
Source: Front Pediatr. 2026 Jan 7;13:1654835. doi: 10.3389/fped.2025.1654835 (PMC12819612; doi:10.3389/fped.2025.1654835)

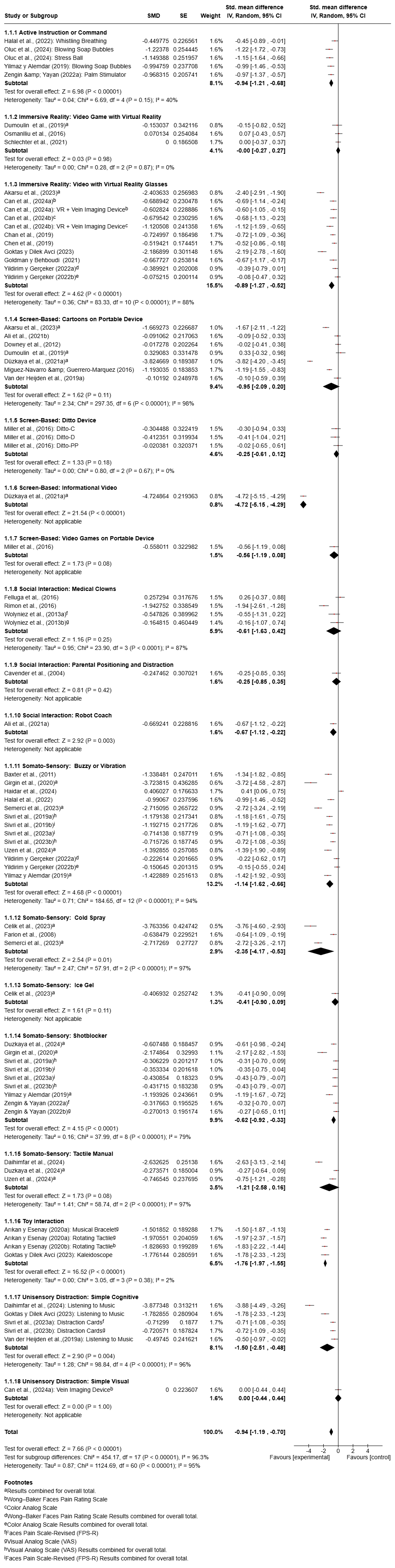

Supplement: Supplementary file 1 [file Image1.png]

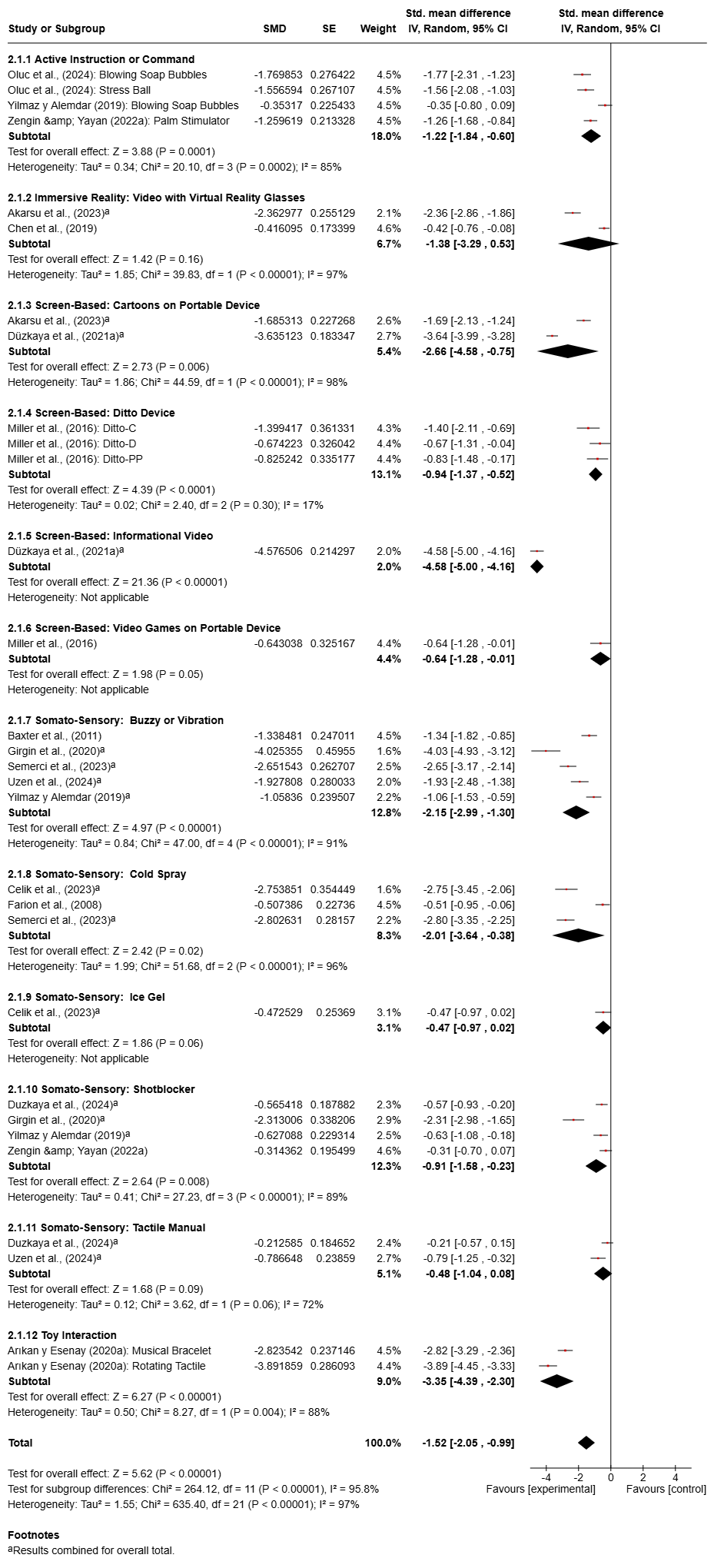

Supplement: Supplementary file 2 [file Image2.png]

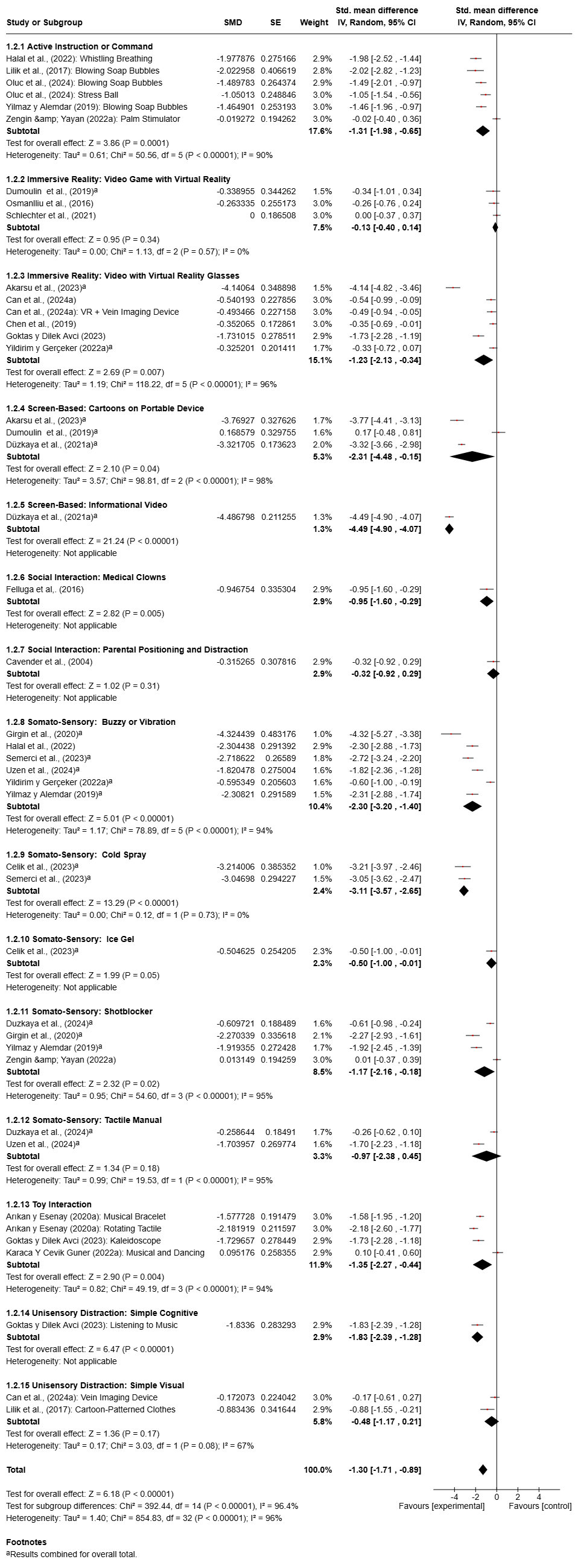

Supplement: Supplementary file 3 [file Image3.png]

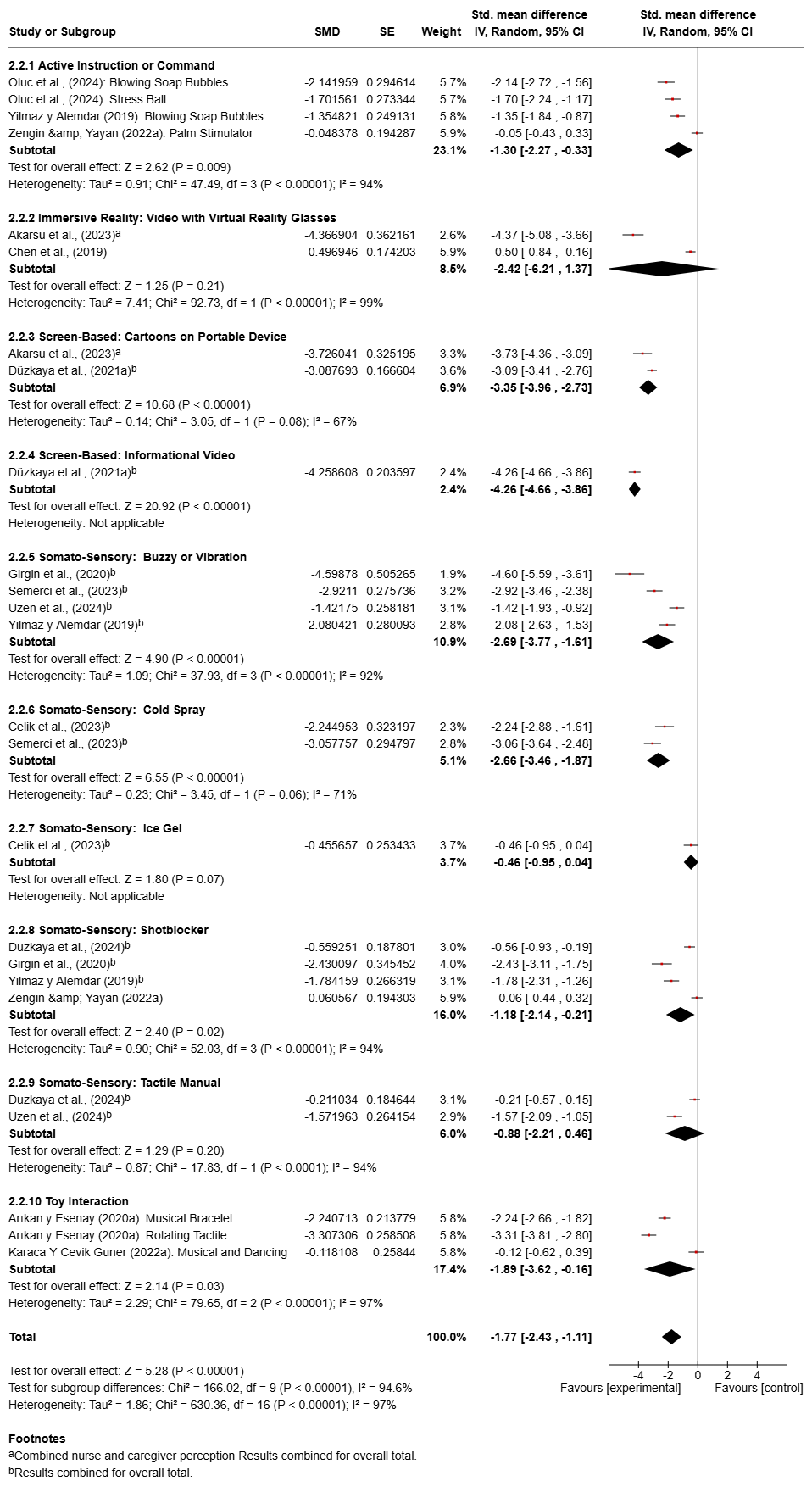

Supplement: Supplementary file 4 [file Image4.png]

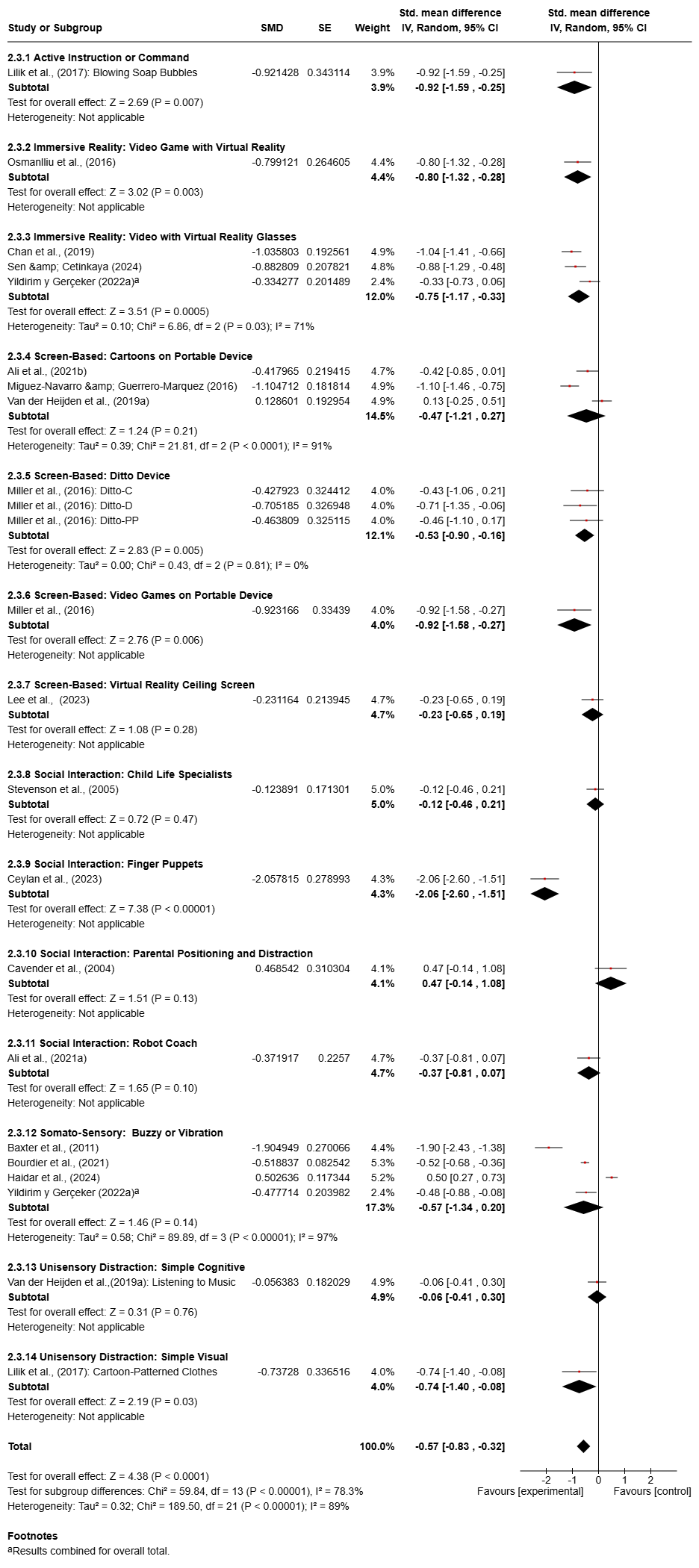

Supplement: Supplementary file 5 [file Image5.png]

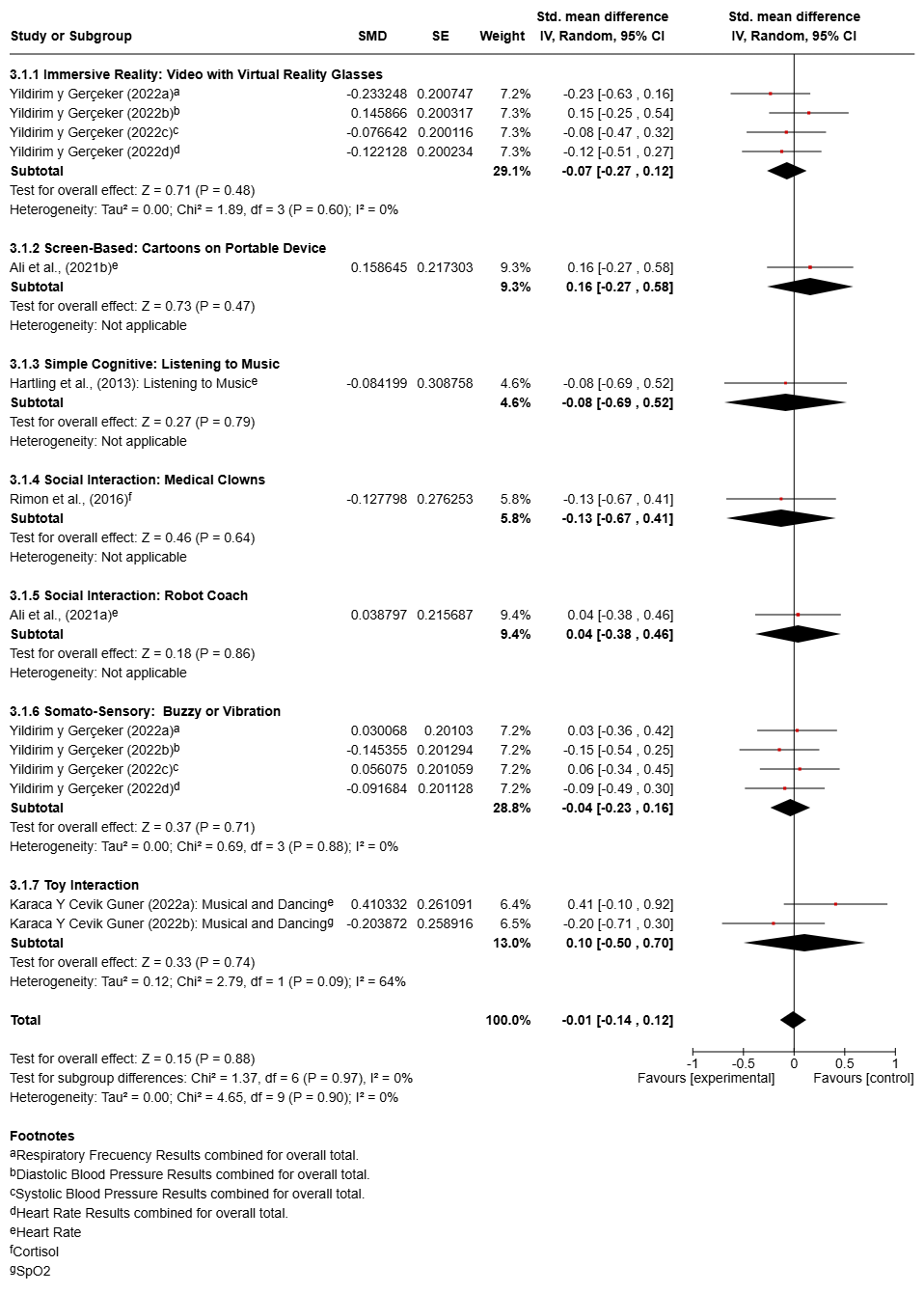

Supplement: Supplementary file 6 [file Image6.png]

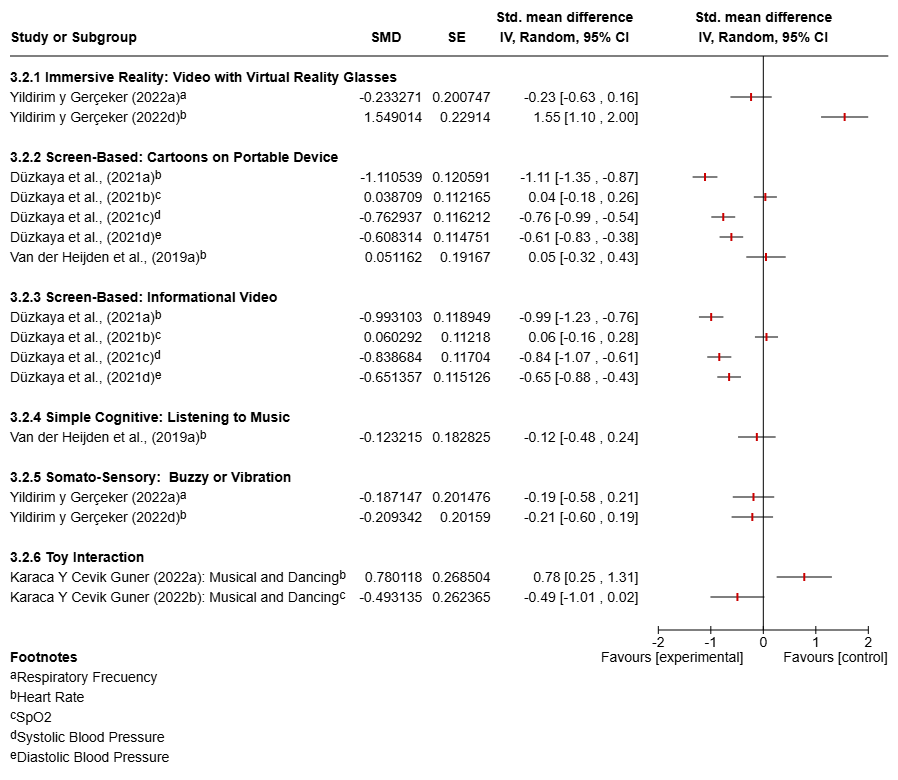

Supplement: Supplementary file 7 [file Image7.png]
