# Supplementary material for: Efficacy of psychosensory interventions for the management of pediatric pain, fear, and distress during emergency care: a systematic review and meta-analysis of randomized clinical trials
Source: Front Pediatr. 2026 Jan 7;13:1654835. doi: 10.3389/fped.2025.1654835 (PMC12819612; doi:10.3389/fped.2025.1654835)
Supplement: Supplementary file 8 [file Table1.docx]

**Supplementary Material 1. Search Strategy**

**Databases searched:** PubMed, PsycINFO, CINAHL, SCOPUS, and Web of Science (WOS).
**Time frame:** January 2004 – September 2024.
**Last search date:** September 30, 2024.

| Database | Platform | Search String | Date of Search |
| --- | --- | --- | --- |
| PubMed | NCBI | ("Child"[Mesh] OR "Child, Preschool"[Mesh] OR "Adolescent"[Mesh] OR "Infant"[Mesh]) AND  ("Psychotherapy"[Mesh] OR "Patient Education as Topic"[Mesh] OR "Early Intervention, Educational"[Mesh] OR "Parents"[Mesh] OR "Caregivers"[Mesh] OR "Distraction" OR "Virtual Reality" OR "Buzzy" OR "Video" OR "Play Therapy" OR "Comfort Positioning" OR "Non-Pharmacological" OR "Relaxation Techniques" OR "Cognitive Intervention" OR "Behavioral Intervention" OR "Sensory Intervention" OR "Mindfulness " OR "Breathing Exercises" OR "Storytelling" OR "Therapeutic Play" OR "Positive Reinforcement" OR "Parental Presence" OR "Child Life Specialist" OR "Bubble Blowing" OR "Book" OR "Puppetry" OR "Medical Play" OR "Cold Spray" OR "Finger Puppet" OR "Medical Clown" OR "Clown" OR "Robot" OR "Music" OR "Toy" OR "Kaleidoscope" OR "Palm stimulator" OR "Shot blocker" OR "Game" OR "Parental Distraction" OR "Parental Coaching" OR "Animal-Assisted Therapy" OR "Therapeutic Dog" OR "Pet Therapy" OR "Tactile Stimulation" OR "Aromatherapy" OR "Headphones" OR "Tablet" OR "Augmented Reality" OR "Wearable Devices")  AND  ("Anxiety"[Mesh] OR "Stress, Psychological"[Mesh] OR "Pain"[Mesh] OR "Fear"[Mesh])  AND  ("Emergencies"[Mesh] OR "Emergency Treatment"[Mesh] OR "Emergency Service, Hospital"[Mesh] OR "Emergency Medical Technicians"[Mesh] OR "Emergency Medical Services"[Mesh] OR "Emergency Services" OR "Emergency Department" OR "Pediatric Guard" OR "Emergency Room" OR "Urgent Care" OR "Acute Care" OR "Pediatric Trauma" OR "Emergency surgery" OR "Immediate Care" OR "Pre-Hospital Care") | September 30, 2024. |
| PsycINFO | EBSCOhost | (MM "Pediatrics" OR MM "Childhood" OR MM "Preschool Age" OR MM "School Age" OR "Child" OR MM "Adolescent")  AND  (DE "Psychoeducation" OR DE "Anxiety Management" OR DE "Behavior Modification" OR DE "Brief Interventions" OR DE "Cognitive Behavior Therapy" OR DE "Cognitive Stimulation Therapy" OR DE "Cognitive Techniques" OR DE "Computer Assisted Therapy" OR DE "Counseling" OR DE "Creative Arts Therapy" OR DE "Mental Health Programs" OR DE "Mind Body Therapy" OR DE "Mindfulness-Based Interventions" OR DE "Movement Therapy" OR DE "Psychotherapy" OR DE "Relaxation Therapy" OR DE "Strengths-Based Interventions" OR DE "Symptoms Based Treatment" OR DE "Educational Therapy" OR DE "Health Education" OR DE "Mental Health Education" OR DE "Cognitive Therapy" OR DE "Cognitive Restructuring" OR DE "Self-Instructional Training" OR DE "Applied Behavior Analysis" OR DE "Behavior Contracting" OR DE "Behavior Therapy" OR DE "Biofeedback Training" OR DE "Contingency Management" OR DE "Self-Management" OR DE "Time Out" OR DE "Acceptance and Commitment Therapy" OR DE "Cognitive Processing Therapy" OR DE "Prolonged Exposure Therapy" OR DE "Affirmative Therapy" OR DE "Analytical Psychotherapy" OR DE "Brief Relational Therapy" OR DE "Emotion Focused Therapy" OR DE "Existential Therapy" OR DE "Experiential Psychotherapy" OR DE "Expressive Psychotherapy" OR DE "Eye Movement Desensitization Therapy" OR DE "Gestalt Therapy" OR DE "Humanistic Psychotherapy" OR DE "Hypnotherapy" OR DE "Individual Psychotherapy" OR DE "Narrative Therapy" OR DE "Rational Emotive Behavior Therapy" OR DE "Reality Therapy" OR DE "Relationship Therapy" OR DE "Solution Focused Therapy" OR DE "Strategic Therapy" OR DE "Coping Behavior" OR "Distraction" OR "Virtual Reality" OR "Buzzy" OR "Video" OR "Play Therapy" OR "Comfort Positioning" OR "Non-Pharmacological" OR "Relaxation Techniques" OR "Mindfulness " OR "Breathing " OR "Storytelling" OR "Play" OR "Positive Reinforcement" OR "Parental Presence" OR "Child Life Specialist" OR "Bubble Blowing" OR "Book" OR "Puppetry" OR "Medical Play" OR "Cold Spray" OR "Ditto" OR "Finger Puppet" OR "Medical Clown" OR "Clown" OR "Robot" OR "Musical Toy" OR "Kaleidoscope" OR "Palm stimulator" OR "Shockbloker" OR "Video Game" OR "Parental Distraction" OR "Parental Coaching" OR "Animal-Assisted Therapy" OR "Therapeutic Dog" OR "Pet Therapy" OR "Tactile Stimulation" OR "Aromatherapy" OR "Headphones" OR "Tablet" OR "Augmented Reality" OR "Wearable Devices")  AND  (MM "Pain" OR MM "Fear" OR MM "Anxiety" OR MM "Distress")  AND  (MM "Emergency Management" OR MM "Emergency Medical Technicians" OR MM "Emergency Medicine" OR MM "Emergency Services" OR "Emergency Department" OR "Pediatric Guard" OR "Emergency Room" OR "Emergency Treatment" OR "Pediatric Emergency" OR "Urgent Care" OR "Trauma Center" OR "Pediatric Urgent Care" OR "Acute Care" OR "Pediatric Trauma" OR "Emergency surgery" OR "Immediate Care" OR "First Responders" OR "Emergency Health Services" OR "Emergency Nursing" OR "Pre-Hospital Care" OR "Pediatric Emergency Response") | September 30, 2024. |
| CINAHL | EBSCOhost | ((MM "Child") OR (MM "Child, Preschool") OR (MM "Adolescence"))  AND  ((MM "Psychoeducation") OR (MM "Psychosocial Intervention") OR (MM "Play Therapy") OR (MM "Pet Therapy+") OR (MM "Music Therapy") OR (MM "Guided Imagery") OR (MM "Hypnosis+") OR (MM "Crisis Intervention") OR (MM "Behavior Modification+") OR (MM "Art Therapy") OR ("Distraction") OR ("Virtual Reality") OR ("Buzzy") OR ("Video") OR ("Comfort Positioning") OR ("Non-Pharmacological") OR ("Relaxation") OR ("Cognitive Behavioral") OR ("Mindfulness") OR ("Breathing") OR ("Storytelling") OR ("Positive Reinforcement") OR ("Parental Presence") OR ("Parental Behavior") OR ("Child Life Specialist") OR ("Bubble Blowing") OR ("Book") OR ("Puppetry") OR ("Play") OR ("Cold Spray") OR ("Ditto") OR ("Finger Puppet") OR ("Medical Clown") OR ("Clown") OR ("Robot") OR ("Toy") OR ("Kaleidoscope") OR ("Palm stimulator") OR ("Shot Blocker") OR ("Game") OR ("Parental Distraction") OR ("Parental Coaching") OR ("Tactile Stimulation") OR ("Aromatherapy") OR ("Headphones") OR ("Tablet") OR ("Augmented Reality") OR ("Wearable Device"))  AND  ((MM "Pain") OR (MM "Fear") OR (MM "Psychological Distress") OR (MM "Anxiety"))  AND  ((MM "Emergencies+") OR (MM "Emergency Care+") OR ("Emergency Department") OR ("Pediatric Guard") OR ("Emergency Room") OR ("Emergency Guard") OR ("Pediatric Urgent Care") OR ("Emergency Response") OR ("Acute Care") OR ("Immediate Care") OR ("Pediatric Trauma") OR ("Emergency surgery") OR ("Pre-Hospital Care") OR (MM "Pediatric Nurse Practitioners+")) | September 30, 2024. |
| SCOPUS | Elsevier | (TITLE-ABS-KEY(( "Preschool" OR "child" OR "adolescent" ))  AND  TITLE-ABS-KEY(("Psychoeducation" OR "Play Therapy" OR "Pet Therapy" OR "Music" OR "Guided Imagery" OR "Hypnosis" OR "Art Therapy" OR "Distraction" OR "Virtual Reality" OR "Buzzy" OR "Video" OR "Comfort Positioning" OR "Non-Pharmacological" OR "Relaxation" OR "Cognitive Intervention" OR "Behavioral Intervention" OR "Sensory Intervention" OR "Mindfulness" OR "Breathing" OR "Storytelling" OR "Positive Reinforcement" OR "Parental Presence" OR "Parental Behavior" OR "Child Life Specialist" OR "Bubble Blowing" OR "Book" OR "Puppetry" OR "Cold Spray" OR "Finger Puppet" OR "Medical Clown" OR "Clown" OR "Robot" OR "Toy" OR "Kaleidoscope" OR "Palm stimulator" OR "Shot Blocker" OR "Game" OR "Parental Distraction" OR "Parental Coaching" OR "Tactile Stimulation" OR "Aromatherapy" OR "Headphones" OR "Tablet" OR "Augmented Reality" OR "Wearable Device"))  AND  TITLE-ABS-KEY(( "Pain" OR "Fear" OR "Distress" OR "Anxiety" ))  AND  TITLE-ABS-KEY(("Emergency Medicine" OR "Emergency Services" OR "Emergency Department" OR "Pediatric Guard" OR "Emergency Room" OR "Emergency Treatment" OR "Emergency Medical Technicians" OR "Emergency Medical Services" OR "Pediatric Emergency Medicine" OR "Urgent Care" OR "Trauma Center" OR "Pediatric Urgent Care" OR "Emergency Response" OR "Acute Care" OR "Pediatric Trauma" OR "Emergency surgery" OR "Immediate Care" OR "Pediatric Critical Care" OR "Emergency Nursing" OR "Pre-Hospital Care"))  AND  TITLE-ABS-KEY("Randomized Controlled Trial" OR "RCT")) | September 30, 2024. |
| Web of Science | Clarivate | "Pediatric" OR "Childhood" OR "Preschool Age" OR "School Age" OR "Child" OR "Adolescent  AND  "Psychoeducation" OR "Coping" OR "Play Therapy" OR "Pet Therapy" OR "Music" OR "Imagery" OR "Hypnosis" OR "Art Therapy" OR "Distraction" OR "Virtual Reality" OR "Buzzy" OR "Video" OR "Relaxation" OR "Cognitive Behavioral" OR "Mindfulness" OR "Breathing" OR "Storytelling" OR "Parental Presence" OR "Parental Behavior" OR "Child Life Specialist" OR "Bubble Blowing" OR "Book" OR "Puppetry" OR "Cold Spray" OR "Finger Puppet" OR "Medical Clown" OR "Clown" OR "Robot" OR "Toy" OR "Kaleidoscope" OR "Palm stimulator" OR "Shot Blocker " OR "Game" OR "Parental Distraction" OR "Parental Coaching" OR "Tactile Stimulation" OR "Aromatherapy" OR "Headphones" OR "Tablet" OR "Augmented Reality" OR "Wearable Device"  AND  "Pain" OR "Fear" OR "Distress" OR "Anxiety"  AND  "Emergency Services" OR "Emergency Department" OR "Pediatric Guard" OR "Emergency Room" OR "Urgent Care" OR "Acute Care" OR "Pediatric Trauma" OR "Emergency surgery" OR "Immediate Care" OR "Pre-Hospital Care" | September 30, 2024. |
